# Reverse taxonomy applied to the *Brachionus calyciflorus* cryptic species complex: Morphometric analysis confirms species delimitations revealed by molecular phylogenetic analysis and allows the (re)description of four species

**DOI:** 10.1371/journal.pone.0203168

**Published:** 2018-09-20

**Authors:** Evangelia Michaloudi, Spiros Papakostas, Georgia Stamou, Vilém Neděla, Eva Tihlaříková, Wei Zhang, Steven A. J. Declerck

**Affiliations:** 1 Department of Zoology, School of Biology, Aristotle University of Thessaloniki, Τhessaloniki, Greece; 2 Division of Genetics and Physiology, Department of Biology, University of Turku, Turku, Finland; 3 Institute of Scientific Instruments, Academy Of Sciences of the Czech Republic, Brno, Czech Republic; 4 Netherlands Institute of Ecology, Department of Aquatic Ecology, Wageningen, The Netherlands; Universitetet i Bergen, NORWAY

## Abstract

The discovery and exploration of cryptic species have been profoundly expedited thanks to developments in molecular biology and phylogenetics. In this study, we apply a reverse taxonomy approach to the *Brachionus calyciflorus* species complex, a commonly studied freshwater monogonont rotifer. By combining phylogenetic, morphometric and morphological analyses, we confirm the existence of four cryptic species that have been recently suggested by a molecular study. Based on these results and according to an exhaustive review of the taxonomic literature, we name each of these four species and provide their taxonomic description alongside a diagnostic key.

## Introduction

Species recognition has been revolutionized by the advent of DNA sequence analysis and barcoding [1, 2]. DNA sequences have helped reveal the existence of cryptic species in a wide variety of taxa [3, 4, 5], and in so doing, have drawn attention to the limitations of traditional species recognition based solely on morphological characteristics. To render species delimitations and descriptions more robust, molecular methods are nowadays increasingly combined with morphology, ecology, cross-fertilization data as well as with other sources of information (e.g., behavioural data, biogeography) in the framework of a taxonomical approach coined as integrative taxonomy [6, 7]. However, even when identified by modern methods, cryptic species often remain taxonomically cryptic [8] because current research practice typically ignores the need of providing a formal description of their diagnostic morphological traits and to suggest a valid taxonomic name. The taxonomic identity of a name is nevertheless fundamental for nomenclatural stability [9, 10, 11]. Unambiguous recognition of cryptic species is further essential for many research purposes, ranging from species diversity inventories and the interpretation of community phylogenetic and biogeographical patterns to the assessment of species’ ecological tolerance or evolutionary potential [12], and the set-up of experiments [13]. To this end, reverse taxonomy consists of an approach that largely uses molecular evidence to guide the morphological description of cryptic species [14]. As such, it provides a powerful tool to discover, describe and assign taxonomically valid names to previously unknown species and thus resolve many of the problems associated to taxonomic crypsis [8, 15].

Rotifera is a phylum of microscopic organisms commonly found in inland waters throughout the world [16] and often harbour high levels of cryptic species diversity [17, 18, 19, 20]. Molecular phylogenetics has proven to be a very effective strategy in recognizing evolutionary significant units of diversity of putative species status in rotifers [18, 21]. An illustrative example of the successful deciphering of a rotifer cryptic species complex is the case of the euryhaline *Brachionus plicatilis* Müller, 1786 [17, 19]. By inspecting molecular phylogenies, at least nine divergent lineages were initially recognized in this taxon [17], which then, by using samples from additional geographic regions, were increased to present-day 15 putative species [19]. Boundaries between most of these species were also confirmed by the results of cross-fertilization experiments [22]). By applying modern phylogenetic species delimitation methods (e.g., reviewed in Fontaneto et al., [23]), the species status of these cryptic species was further supported in a recent study [19]. Such molecular evidence combined with morphometric analysis has also been fundamental to the taxonomic description of some of these species [24, 25, 26].

Of all freshwater monogonont rotifers, *Brachionus calyciflorus* Pallas, 1766 is probably the most widely studied in ecology (e.g. [27, 28]), evolutionary biology (e.g. [29, 30, 12]), ecotoxicology (e.g. [31, 32, 33]), and aquaculture (e.g. [34, 35]). Indicative of its importance, *B*. *calyciflorus* was the first monogonont rotifer of which the genome was published [36]. Nevertheless, its taxonomy remains particularly confused. Since *B*. *calyciflorus* served as type species for the *Brachionus* genus [37], numerous morphological variants on subspecies and infrasubspecific level have been described (e.g., [38, 39]). This situation has led [40] to express the need for a thorough revision of the taxon and the validation of these numerous variants recorded worldwide in ecological studies (Table A in S1 Text). A lot of the confusion undoubtedly arises from its high phenotypic variability [37]. Recently, the taxon was suggested to consist of a cryptic species complex [41, 42]. By applying an integrative taxonomy approach, Papakostas et al. [20] coupled the analysis of a large dataset of DNA sequences with morphometric data. The molecular analyses revealed frequent cases of discordant patterns between the mitochondrial cytochrome *c* oxidase subunit I (COI) and nuclear internal transcribed spacer 1 (ITS1) markers, a phenomenon often described as mitonuclear discordance. Combined with microsatellite analysis, the possibility of hybrid introgression between four cryptic species of the *B*. *calyciflorus* complex, coded as ‘A’, ‘B’, ‘C’, and ‘D’, was suggested [20]. In light of this evidence, an intriguing observation was that the nuclear marker ITS1 was found to be a more reliable predictor of the species than the mitochondrial marker COI as it explained a much higher proportion of the morphometric variation (71% vs. 36%), with the variation explained by COI being also entirely redundant to that explained by ITS1 [20].

Papakostas et al. [20] mainly studied the morphometry of two of the four species, those coded as ‘B’ and ‘C’. In this study, we aim at comprehensively studying the morphometry and morphology of all four *B*. *calyciflorus* cryptic species recognized by Papakostas et al. [20]. For this purpose, we collected and cultured additional rotifer clones representing multiple populations for each of the four suggested species. We then measured several morphometric traits under standardized conditions and tested for the existence of morphometric differentiation among each of the four species pairs. We additionally proceeded to an elaborate morphological investigation, which entailed an exhaustive review of the taxonomic literature, comparing the morphology of the groups with previously published descriptions of forms and variants within the taxon. This effort has resulted in the description of two new species, *B*. *elevatus* sp. nov. and *B*. *fernandoi* sp. nov., and the redescription of two earlier described taxa, *B*. *calyciflorus* Pallas, 1766 and *B*. *dorcas* Gosse, 1851. Furthermore, we provide a key to guide the identification of the species using diagnostic morphological traits. We also provide in the supplementary materials an organized collection of ITS1 sequences available in GenBank that may be used to facilitate identification of each of these species using this particular molecular marker. Altogether, this study resolves the taxonomic identity of a much-studied freshwater monogonont rotifer species complex, it suggests distinct valid names that should be used to refer to each of these species, and provides morphological and genetic resources to facilitate the recognition and thus study of each of these species.

## Material and methods

### Resting egg collection and clone line establishment

Surficial sediment samples were collected from lakes and ponds in The Netherlands (Table 1). All samples analysed for this study were taken from public waterways in The Netherlands and therefore do not require permission. The field studies did not involve endangered or protected species. *Brachionus* sp. resting eggs were separated using the sugar flotation method [43]. Resting eggs hatched under continuous light in Petri dishes with distilled water. The dishes were checked at 12-hour intervals, and hatchlings were removed when present. Clonal lines were initially established by individually transferring hatched *Brachionus calyciflorus* females from the dishes into wells of a 24-well plate filled with chemostat grown *Chlamydomonas reinhardtii* (1000 μmol C L^-1^). After clonal lines had established, cultures were upscaled by transferring the populations into 20 mL coulter cups (Beckman Coulter®) with 8 mL of food. These populations served as stock cultures, were maintained at room temperature (22–24°C) under continuous light and supplied with fresh medium and food every two days. After a few months of growth in the lab, these stock cultures served as the source of individuals for morphometric analysis.

**Table 1:** Overview of the materials used for each of the species described in this study. nuITS1 delimitation: Putative species as suggested by [20]; coordinates are WGS84.

nuITS1 delimitation	Species	Population ID	Clone ID	n	Data origin	Latitude	Longitude	Sampling time
‘A’	*B*. *dorcas*	NL7	J	10	Papakostas et al. [20]	N 51.854065°	E 5.893175°	Jun. 2011
		NL68	C01	13	present study	N 52.083253°	E 4.323353°	Apr. 2016
		NL68	C09	17	present study	N 52.083253°	E 4.323353°	Apr. 2016
		NL68	C10	18	present study	N 52.083253°	E 4.323353°	Apr. 2016
		NL128	C25	17	present study	N 52.640324°	E 4.730287°	Mar. 2014
		NL128	C30	10	present study	N 52.640324°	E 4.730287°	Mar. 2014
		NL129	C21	13	present study	N 52.652613°	E 4.776304°	Mar. 2014
		NL129	C43	13	present study	N 52.652613°	E 4.776304°	Mar. 2014
		NL134	C01	16	present study	N 52.734063°	E 4.883798°	Mar. 2014
		NL134	C02	11	present study	N 52.734063°	E 4.883798°	Mar. 2014
		NL134	C04	13	present study	N 52.734063°	E 4.883798°	Mar. 2014
‘B’	*B*. *elevatus* sp. nov.	NL7	B	19	Papakostas et al. [20]	N 51.854065°	E 5.893175°	Jun. 2011
		NL7	C24	14	present study	N 51.854065°	E 5.893175°	Apr. 2016
		NL7	E	17	Papakostas et al. [20]	N 51.854065°	E 5.893175°	Jun. 2011
		NL7	G	20	Papakostas et al. [20]	N 51.854065°	E 5.893175°	Jun. 2011
		NL67	E	18	Papakostas et al. [20]	N 52.080972°	E 4.313722°	Aug. 2012
		NL69	B	20	Papakostas et al. [20]	N 52.090694°	E 4.338444°	Aug. 2012
		NL69	C25	17	present study	N 52.090694°	E 4.338444°	Apr. 2016
		NL69	D	19	Papakostas et al. [20]	N 52.090694°	E 4.338444°	Aug. 2012
		NL69	G	19	Papakostas et al. [20]	N 52.090694°	E 4.338444°	Aug. 2012
		NL69	H	19	Papakostas et al. [20]	N 52.090694°	E 4.338444°	Aug. 2012
		NL69	K	20	Papakostas et al. [20]	N 52.090694°	E 4.338444°	Aug. 2012
		NL69	L	18	Papakostas et al. [20]	N 52.090694°	E 4.338444°	Aug. 2012
		NL69	M	18	Papakostas et al. [20]	N 52.090694°	E 4.338444°	Aug. 2012
		NL69	R	16	Papakostas et al. [20]	N 52.090694°	E 4.338444°	Aug. 2012
‘C’	*B*. *calyciflorus* s.s.	NL7	C01	20	present study	N 51.854065°	E 5.893175°	Apr. 2016
		NL7	K	12	Papakostas et al. [20]	N 51.854065°	E 5.893175°	Jun. 2011
		NL7	R	2	Papakostas et al. [20]	N 51.854065°	E 5.893175°	Jun. 2011
		NL22	A	17	Papakostas et al. [20]	N 51.985263°	E 5.665691°	Jul. 2012
		NL22	D	20	Papakostas et al. [20]	N 51.985263°	E 5.665691°	Jul. 2012
		NL22	F	19	Papakostas et al. [20]	N 51.985263°	E 5.665691°	Jul. 2012
		NL22	K	14	Papakostas et al. [20]	N 51.985263°	E 5.665691°	Jul. 2012
		NL67	B	14	Papakostas et al. [20]	N 52.080972°	E 4.313722°	Aug. 2012
		NL69	C	12	Papakostas et al. [20]	N 52.090694°	E 4.338444°	Aug. 2012
		NL69	E	14	Papakostas et al. [20]	N 52.090694°	E 4.338444°	Aug. 2012
		NL69	F	4	Papakostas et al. [20]	N 52.090694°	E 4.338444°	Aug. 2012
		NL128	C01	17	present study	N 52.640324°	E 4.730287°	Mar. 2014
		NL168	C01	18	present study	N 51.491438°	E 4.306801°	Mar. 2014
‘D’	*B*. *fernandoi* sp. nov.	NL128	C05	18	present study	N 52.640324°	E 4.730287°	Mar. 2014
		NL128	C06	17	present study	N 52.640324°	E 4.730287°	Mar. 2014
		NL129	C01	7	present study	N 52.652613°	E 4.776304°	Mar. 2014
		NL129	C02	19	present study	N 52.652613°	E 4.776304°	Mar. 2014
		NL129	C42	16	present study	N 52.652613°	E 4.776304°	Mar. 2014
		NL129	C44	16	present study	N 52.652613°	E 4.776304°	Mar. 2014
		NL181	C02	15	present study	N 51.839032°	E 4.144425°	Feb. 2014
		NL181	C03	9	present study	N 51.839032°	E 4.144425°	Feb. 2014
		NL181	C07	8	present study	N 51.839032°	E 4.144425°	Feb. 2014
		NL181	C10	11	present study	N 51.839032°	E 4.144425°	Feb. 2014

Species: species names given by current study; n: number of individuals analysed for the morphometric analysis.

### Molecular species identification

To identify the species of the additional clonal cultures used in this study, we employed an extensive database of species-delimited ITS1 unique sequences, called haplotypes, available in Papakostas et al. [20] (Appendix 2: S2 and S3 Tables; available at: http://datadryad.org/resource/doi:10.5061/dryad.8rc4r). For the sake of continuity, we will henceforth use the term, haplotype, to describe unique sequences of ITS1. DNA was extracted from single rotifers, and the ITS1 region was PCR-amplified as described in Papakostas et al. [20]. Amplicons were then Sanger-sequenced to both primer directions by Macrogen Europe (Amsterdam, The Netherlands), and sequencing chromatograms were aligned and manually inspected with Sequencher 4 (Gene Codes). Double peaks and length variance that may indicate heterozygotes was noted and co-occuring sequences were distinguished as described in Papakostas et al. [20]. ITS1 sequences were then appended to the ITS1 haplotypes of Papakostas et al. [20] (available within the file “1_Alignments.zip” through the Dryad repository: http://datadryad.org/resource/doi:10.5061/dryad.8rc4r), and the whole dataset was re-aligned using the mlocarna function of the LocARNA v. 1.9.1 tool [44] with default settings. The alignment was reduced to contain only the complete ITS1 region and identical sequences, referred to as haplotypes, were recognized with DNAsp v. 5 [45]. Newly sequenced clonal cultures with same ITS1 haplotypes as in Papakostas et al. [20] were assigned to the species that has been already deduced. For newly discovered haplotypes, a simple delimitation analysis was run using the generalized mixed-Yule coalescent model (GMYC; [46] as described in Papakostas et al. [20]. The species of these new ITS1 haplotypes was that of already analyzed ITS1 haplotypes by Papakostas et al. [20] grouped in the same species category suggested by the GMYC analysis.

### Morphometry

**Fig 1:**
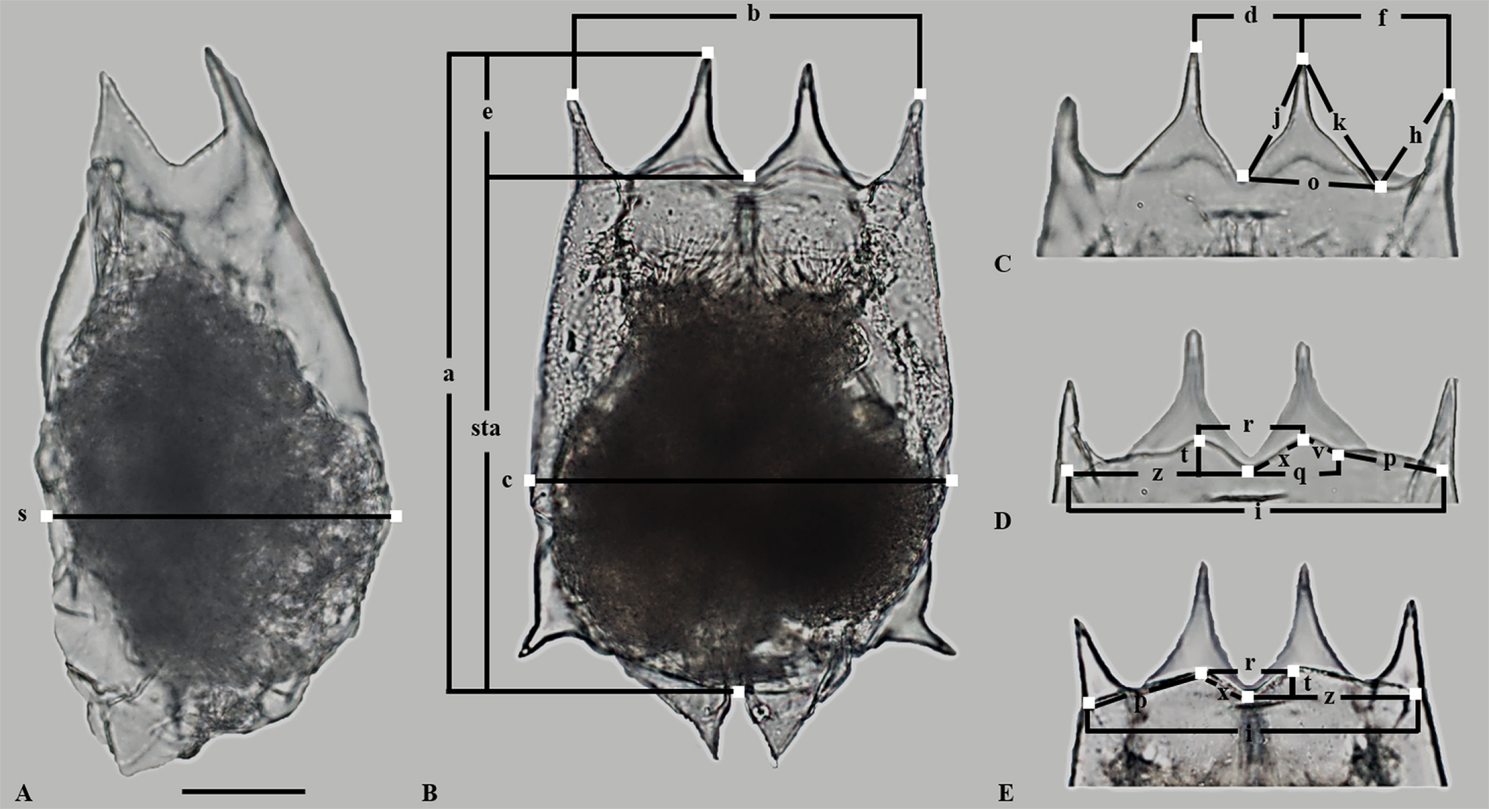
Microphotographs of a *Brachionus calyciflorus* individual. A and B show the major lorica dimensions measured, C the dimensions measured of the anterodorsal margin, D and E the dimensions measured of the anteroventral margin.

Morphometric analysis was performed on formalin-fixed females (4% formalin). The 724 individuals used for the analysis were isolated from 48 clonal cultures (23 of them established by Papakostas et al. [20]). Twenty, whenever possible (range 2–20 Table 1), randomly picked individuals from each clonal culture were examined under a LeitzLaborlux S optical microscope. Microphotographs for each individual were taken with an adjusted camera Canon Power shot A650 IS, and morphometric measurements were obtained using ImageJ [47]. A total of 19 lorica dimensions (Fig 1) were measured based on Fu et al. [48], Ciroz-Perez et al. [24], Proios et al. [49], and Michaloudi et al. [26] with additional measurements made on the anterodorsal and anteroventral side. Two measurements of the anterodorsal side, namely ‘d’ and ‘f’, were not included in the further analysis due to high distortion of the placement of the anterior spines during preservation. All the rotifer microphotographs analyzed for Papakostas et al. [20] are available within the file “9_Rotifer_microphotographs.zip” through the Dryad repository: http://dx.doi.org/10.5061/dryad.8rc4r. All the additional rotifer microphotographs analyzed for the present study are publicly available via the online Dryad repository (accession link: http://datadryad.org/review?doi=doi:10.5061/dryad.4ng70).

### Taxonomy

In order to investigate the taxonomic status of *B*. *calyciflorus* variants since its description (1766) a literature review was conducted until 2016 using Google scholar search engine and the following synonyms as keywords i.e. “*Arthracanthus biremis*”, “*Arthracanthus quadriremis*”, “*Anuraea palea”*, “*Anuraea divaricata”*, “*Brachionus calyciflorus borgerti*”, “*Brachionus calyciflorus amphiceros*”, “*Brachionus calyciflorus anuraeiformis*”, “*Brachionus calyciflorus calyciflorus*” “*Brachionus calyciflorus dorcas*”, “*Brachionus calyciflorus giganteus*”, “*Brachionus calyciflorus spinosus*”, “*Brachionus decipiens*”, “*Brachionus margoi*”, “*Brachionus pala*”, “*Brachionus pala mucronatus*”, “*Brachionus palea*” and “*Brachionus pentacanthus*”. Only studies indicating the existence of at least one of the above species in a zooplankton community was included. In the cases where the same dataset was included in more than one articles, all the articles were included. ‘Grey’ literature (i.e. conference proceedings, theses) was avoided when there was a peer-reviewed published source. For each case, when possible, we provide the specific name of the species, the country, and the area, lake or river in which it was recorded together with the reference source (Table A in S1 Text).

All taxonomic information provided (i.e. spelling, authors names, synomys) in the text have been verified with the List of Available Names [9, 50] and the Rotifer World Catalog [51]

### Nomenclatural acts

The electronic edition of this article conforms to the requirements of the amended International Code of Zoological Nomenclature (ICZN), and hence the new names contained herein are available under that Code from the electronic edition of this article. This published work and the nomenclatural acts it contains have been registered in ZooBank, the online registration system for the ICZN. The ZooBank LSIDs (Life Science Identifiers) can be resolved and the associated information viewed through any standard web browser by appending the LSID to the prefix “http://zoobank.org/”. The LSID for this publication is: urn:lsid:zoobank.org:pub: 73FED4F9-11F0-43C0-9AD0-8D4C14CAE1D3. The electronic edition of this work was published in a journal with an ISSN, and has been archived and is available from the following digital repositories: PubMed Central, LOCKSS.

### Scanning Electron Microscopy (SEM)

#### Full-body

Environmental scanning electron microscopy (ESEM) was applied to each clone using custom modified Quanta 650 FEG microscope equipped with and a non-commercial high-efficiency detector for low dose imaging [52]. Rotifers of each clone were pipped into 5 μL drop of distilled water to clean their surface prior the observation. Live animals were individually transferred on a cooling Peltier stage in the microscope and observed using the Low Temperature Method for ESEM (LTM) [53] that was originally developed for the study of plant samples [54] and optimised for the samples of the present study. Additional purging process of water vapour was applied during LTM to ensure higher relative humidity in a specimen chamber of the ESEM to prevent a sample collapse before its low temperature stabilisation. Samples were observed under following conditions: temperature of the cooling stage was -20°C, air pressure in the ESEM specimen chamber was 200 Pa, beam energy of 10 keV and beam current bellow 20 pA.

#### Trophi

Trophi SEM stub mounts were prepared for clones of each of the four species based on the methodology described by De Smet [55]. Five 50 μL beads of distilled water were pipetted onto a glass slide. A total of six individual rotifers were pipetted into the first 50 μL drop of distilled water, and 10 μL of domestic bleach (3% sodium hypochlorite solution) was added. Animals were observed under a dissecting microscope. Once all trophi had been expelled from the lorica, they were pipetted between the remaining four 50 μL beads of distilled water. Each individual trophi was finally picked up in 1 μL of distilled water and pipetted onto a round coverslip of 12 mm diameter. The pipette tip was changed after each transfer. Once all of the trophi had been transferred, they were left on the bench to dry. Once desiccated, the round coverslip was stuck to a SEM stub that had been prepared with an adhesive tape; the sample was then sputter coated with gold using Agar Sputter Coater. Photographs were taken by JSM-6300 Scanning Electron Microscope.

### Statistical analysis

We applied principal components analysis (PCA) to explore for systematic morphometric differences among the hypothetical species. Redundancy analysis (RDA) was then used to formally test for statistical differences among species. Furthermore, to identify which combinations of morphometric traits discriminate best between species we applied stepwise discriminant function analysis (DFA). Applying DFA on all four species revealed a particularly strong differentiation between species ‘A’ and the other three species, potentially overwhelming differences among the species ‘B’, ‘C’, and ‘D’; thus in order to discriminate the rest of the species as well DFA was applied on two different datasets: (a) including data of all four species and (b) including only the data of species ‘B’, ‘C’ and ‘D’. To account for the unequal representation of species in the dataset, the weight of species was adjusted according to prior probabilities. To assess the robustness of the DFA model, we applied the leave-one-out cross-validation approach. Finally, for each of the individual morphometric variables, we tested for the significance of differences among the four species using ANOVA combined with Turkey post hoc test.

Data were standardized prior to PCA, RDA and DFA statistical analysis and log(x+1) transformed prior to ANOVA. RDA and ANOVA were performed on clonal averages to avoid inflated type I error whereas PCA and DFA were based on data of individual rotifers. PCA and RDA were performed using the function *rda* of the package “vegan” [56] in R. DFA and ANOVA were performed using IBM SPSS Statistics 22 [57].

## Results

### Molecular species identification

All clonal cultures used in this study were categorized to one of the four *B*. *calyciflorus* species previously recognized in Papakostas et al. [20], and no evidence for a new species was found (Table 1; supplementary material of [20]). All but one of the newly generated ITS1 sequences were identical to one of the ITS1 haplotypes previously reported by Papakostas et al. [20] (supplementary material of [20]), which suggests that a large portion of the ITS1 polymorphism has been already covered for the studied geographic region. Only four genotypes were heterozygotes for ITS1 (supplementary material of [20]). Newly generated ITS1 sequences were submitted to GenBank (accession numbers: MF776636-MF776664), while ITS1 alignment, sequencing information and other information relevant to the species delimitation are freely available via the online Dryad repository (accession link: http://datadryad.org/review?doi=doi:10.5061/dryad.4ng70).

### Morphometry

**Fig 2:**
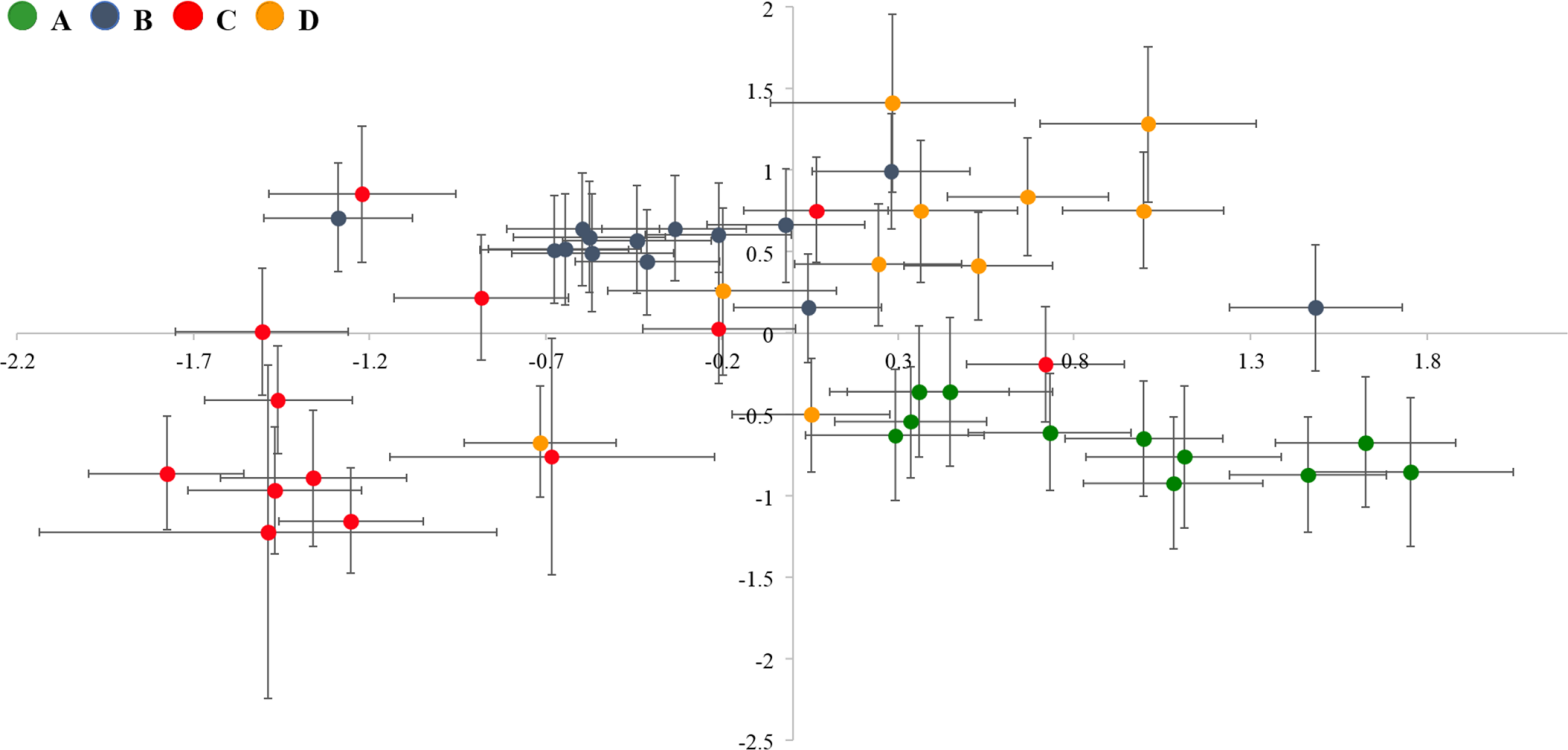
Analysis of the association between molecular species delimitation and morphometry. Representation of sample score averages of each of the studied clones along the first two axes (axis x: PC1, axis y: PC2) of the principal components analysis (PC1 explained 57.67% and PC2 explained 14.74% of the recorded variation) performed on standardized morphometric data. Error bars represent variation between individuals of the same clone (twice the standard error of the mean).

Principal Components analysis positioned the clones into separate clusters that corresponded well with the species delimitation as suggested by Papakostas et al. [20] (Fig 2). The first two axes represented 72.41% (PC1 57.67%, PC2 14.74%) of the total observed variation. According to RDA, the factor ‘species’ explained 53% of the total morphometric variation (F: 16.861; P < 0.001). Additional tests showed significant differences among each pair of species with R^2^-values ranging between 19 and 55% (S1 Table).

**Fig 3:**
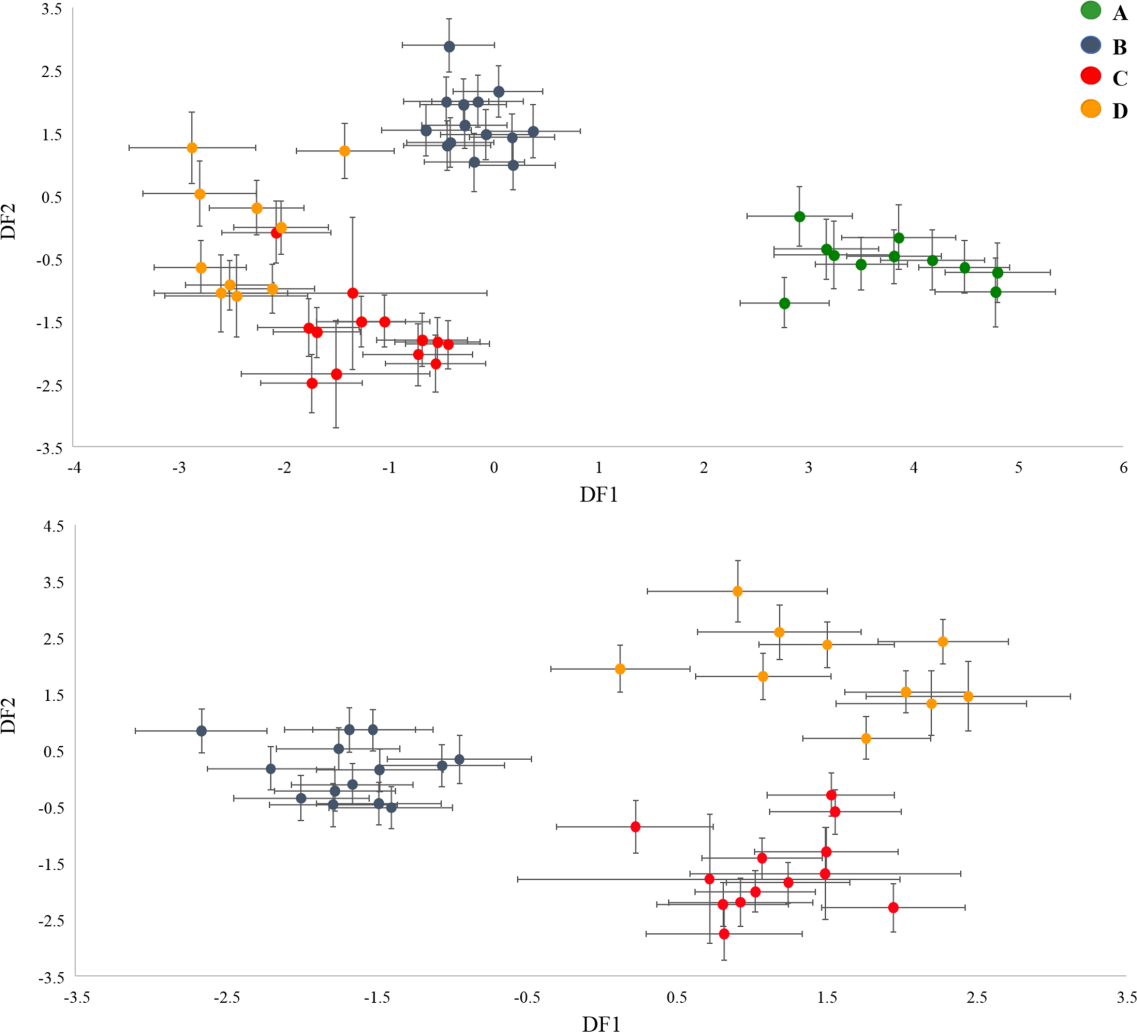
Analysis of the association between molecular species delimitation and morphometry. Scatterplot showing the discrimination of species groups based on the canonical discriminant functions of the discriminant analysis performed on all measured individuals of the studied clones (upper) for species ‘A’, ‘B’, ‘C’, ‘D’ and (lower) for species ‘B’, ‘C’, ‘D’. Error bars represent variation between individuals of the same clone (twice the standard error of the mean).

The range of the dimensions measured for the four species is shown in S2 Table. In agreement with the RDA analysis, most of the variables showed statistically significant differences among the four groups, despite a large overlap in values (Table 2, S2 Table). Tables 3 and 4 show the results of the discriminant analysis. When applied to all four species Classification Function I accounted for 58.5% of the total variance (Table 3) and clearly distinguished the species previously reported as ‘A’ from the rest of the species (Fig 3A). The variables weighing the most in this function were traits from the anteroventral margin, namely ‘q’, ‘v’, ‘r’ and ‘i’, traits representing overall body size (i.e. standard length ‘sta’ and width ‘c’), and the length of the anterodorsal spines ‘h’ and ‘k’ (Table 3). Species ‘B’, ‘C’ and ‘D’ were separated from each other when DFA was applied to these three species only (Fig 3B). In this case Classification Function I accounted for 57.2% of the total variance (Table 4) and distinguished species ‘B’ from species ‘C’ and ‘D’. The variable weighting the most in this function were traits from the anteroventral margin, namely ‘q’ and ‘v’ and body width ‘c’. Classification Function II accounted for 42.8% (Table 4) of the variance and mainly differentiated between species ‘C’ and ‘D’ (Fig 3B). The variable weighting the most in this function was the distance of the lateralanterodorsal spines ‘b’ and the width of the medial sinus of the anteroventral margin ‘r’. Cross-validation of the individuals’ classification based on the classification functions (S3 and S4 Tables) of the discriminant analysis correctly identified 96% of ‘A’ species, 95.7% of ‘B’ species, 90.7% of ‘C’ species and 88.2% of ‘D’ species individuals, suggesting that the identification of species based on morphometric variables might be possible with reasonable success.

**Table 2:** Results of ANOVA and Turkey post-hoc test for differences in lorica traits between the four species of the *B*. *calyciflorus* cryptic species complex.

	Bonferroni post-hoc test				ANOVA		
Measurement	‘A’	‘B’	‘C’	‘D’	df	F	*p*
s	a	b	c	a	3	138.13	<0.0001
c	a	b	c	d	3	143.38	<0.0001
e	a	b	c	b	3	223.68	<0.0001
b	a	b	bd	d	3	72.27	<0.0001
o	a	b	b	d	3	54.18	<0.0001
h	a	b	b	d	3	76.44	<0.0001
k	a	b	c	d	3	187.06	<0.0001
j	a	b	c	b	3	217.83	<0.0001
t	a	b	c	a	3	135.89	<0.0001
i	a	b	c	d	3	142.75	<0.0001
p	a	b	c	bc	3	109.86	<0.0001
v	bd	b	c	d	3	28.49	<0.0001
x	a	b	c	d	3	146.10	<0.0001
z	a	b	c	d	3	152.93	<0.0001
q	a	a	c	a	3	72.76	<0.0001
r	a	b	c	d	3	128.70	<0.0001
sta	a	b	c	a	3	83.39	<0.0001

Species with same letters did not differ significantly.

**Table 3:** Stepwise discriminant analysis based on the morphometric data of species ‘A’, ‘B’, ‘C’ and ‘D’ of the *B*. *calyciflorus* species complex.

	Function 1		Function 2		Function 3	
Measurement	Coefficient	Correlation	Coefficient	Correlation	Coefficient	Correlation
s	-.488	.102	-.202	.050	.622	.646
c	.912	.290	.485	.133	-.650	.497
e	.364	.401	.186	.054	.238	.506
b	.165	.235	-.492	-.219	-.577	.292
o	-.269	.200	-.104	-.112	-.410	.278
h	-.884	.152	-.498	-.097	.093	.424
k	.564	.344	-.216	-.061	.387	.557
j	.454	.402	.345	.051	-.099	.493
t	-.113	.024	.429	.443	-.476	-.012
i	.819	.302	-.276	.007	-.448	.570
p	.526	.240	.897	-.181	.579	.212
v	-1.346	.026	-.785	.102	.713	.158
x	-.264	-.041	.457	.437	.091	.498
z	-.199	.299	-.745	.017	.500	.607
q	1.871	.082	1.858	.252	-.298	.197
r	-.813	-.097	.419	.324	.566	.592
sta	-.810	.133	-.286	-.005	.470	.568
Eigenvalue	4.291		1.794		1.248	
% variance	58.5		24.5		17.0	

Values of the three canonical functions are shown. Coefficient: standardized coefficient for the canonical discriminant function. Correlation: pooled within group correlation coefficient between the body measurement and the canonical discriminant function.

**Table 4:** Stepwise discriminant analysis based on the morphometric data of species ‘B’, ‘C’ and ‘D’ of the *B*. *calyciflorus* species complex.

	Function 1		Function 2	
Measurement	Coefficient	Correlation	Coefficient	Correlation
s	.650	.118	.695	.544
c	-1.127	-.0.97	-.657	.398
e*		-.054		.328
b	.035	.167	-.829	.0.60
o	.102	.086	-.281	.114
h	.918	.140	.136	.249
k*		.080		.299
j	-.792	-.087	.198	.333
t	-.499	-.389	-.129	.213
i*		.047		.407
p*		.121		-.019
v	2.093	-.044	-.104	.162
x	-.072	-.187	.261	.643
z*		.026		.400
q	-2.293	-.174	.063	.248
r	.229	-.036	.803	.684
sta	.794	.122	.411	.429
Eigenvalue	2.247		1.683	
% variance	57.2		42.8	

Values of the three canonical functions are shown. Coefficient: standardized coefficient for the canonical discriminant function. Correlation: pooled within group correlation coefficient between the body measurement and the canonical discriminant function.

* this variable not used in the analysis

The redundancy and discriminant function analyses provide robust formal statistical support for the existence of four different morphometric groups that correspond strongly to the evolutionary units as proposed by Papakostas et al. [20]. Our detailed morphological investigation confirms these conclusions and results in the redescription of two formerly described species, *B*. *calyciflorus* Pallas, 1766 and *B*. *dorcas* Gosse, 1851, and the description of two new species, *B*. *elevatus* sp. nov. and *B*. *fernandoi* sp. nov. These species correspond to the evolutionary units that were revealed by Papakostas et al. [20] based on nuITSI sequence data: species ‘A’: *B*. *dorcas*; species ‘B’: *B*. *elevatus* sp. nov.; species ‘C’: *B*. *calyciflorus* sensu stricto (s.s.); species ‘D’: *B*. *fernandoi* sp. nov.

### Redescription of *Brachionus calyciflorus* s.s. Pallas, 1766

#### Taxonomy

Class: Eurotatoria De Ridder, 1957
Subclass: Monogononta Plate, 1889
Superorder: Pseudotrocha Kutikova, 1970
Order: Ploima Hudson & Gosse, 1886
Family: Brachionidae Ehrenberg, 1838
*Brachionus calyciflorus* Pallas, 1766

*Brachionus calyciflorus* Pallas 1766 [58], p. 96,

*Brachionus palea*, Ehrenberg 1830 [59], p. 68,

*Anuraea palea*, Ehrenberg 1830 [59], p.68

*Brachionus amphiceros*, Ehrenberg 1838 [60], p. 511, pl 63, Fig 2

*Brachionus calyciflorus* var. *amphiceros*, Ehrenberg 1838 [60]

*Brachionus pala*, Ehrenberg 1838 [60], p. 511, pl 63, Fig 1, pl 50, Fig 2

*Anuraea divaricata*, Weisse 1845 [61], p. 142, pl 2, Fig 13–14

*Arthracanthus biremis*, Schmarda 1854 [62], p. 22, pl 6, Fig 5

*Arthracanthus quadriremis*, Schmarda 1854 [62], p.12, pl 5, Fig 1

*Brachionus margoi*, Daday 1883 [63], p. 290

*Brachionus decipiens*, Plate 1886 [64], p 73

*Brachionus pentacanthus*, France 1894 [65], p. 172, pl 5, Figs 3 and 4

*Brachionus pala anuraeiformis*, Brehm 1909 [66], p. 308, Fig 1

*Brachionus calyciflorus* f. *anuraeiformis*, Brehm 1909 [66], p. 210, text-Fig

*Brachionus pala mucronatus*, Spandl 1922 [67], p. 275, text-Fig.

#### Etymology

The name ‘calyciflorus’ originates from the latin words ‘calyx’ (originally coming from the greek ‘κάλυξ’) and ‘flos’ meaning flower. Probably it was used due to the resemblage of *B*. *calyciflorus* to the shape of a flower-calyx.

#### Material examined

A total of 183 individuals were examined coming from 13 clones established from resting eggs collected in 6 water bodies from The Netherlands (Table 1).

Permanent glycerin glass slide mounts, each containing a single specimen, were prepared according to Jersabek et al. [68], and deposited in the Frank J. Myers collection at the Academy of Natural Sciences in Philadelphia (ANSP) with catalogue numbers ANSP [2100–2104].

Based on the literature review (S1 Text) and the information available in the List Available Names (LAN) [9, 50] we conclude that no type material of *B*. *calyciflorus* is available. Following the guidelines of ICZN, since we are dealing with a species complex, we decided to designate a specific slide as neotype.

Neotype: A parthenogenetic female in a permanent glycerin glass with catalogue number [2100]

#### Description

**Fig 4:**
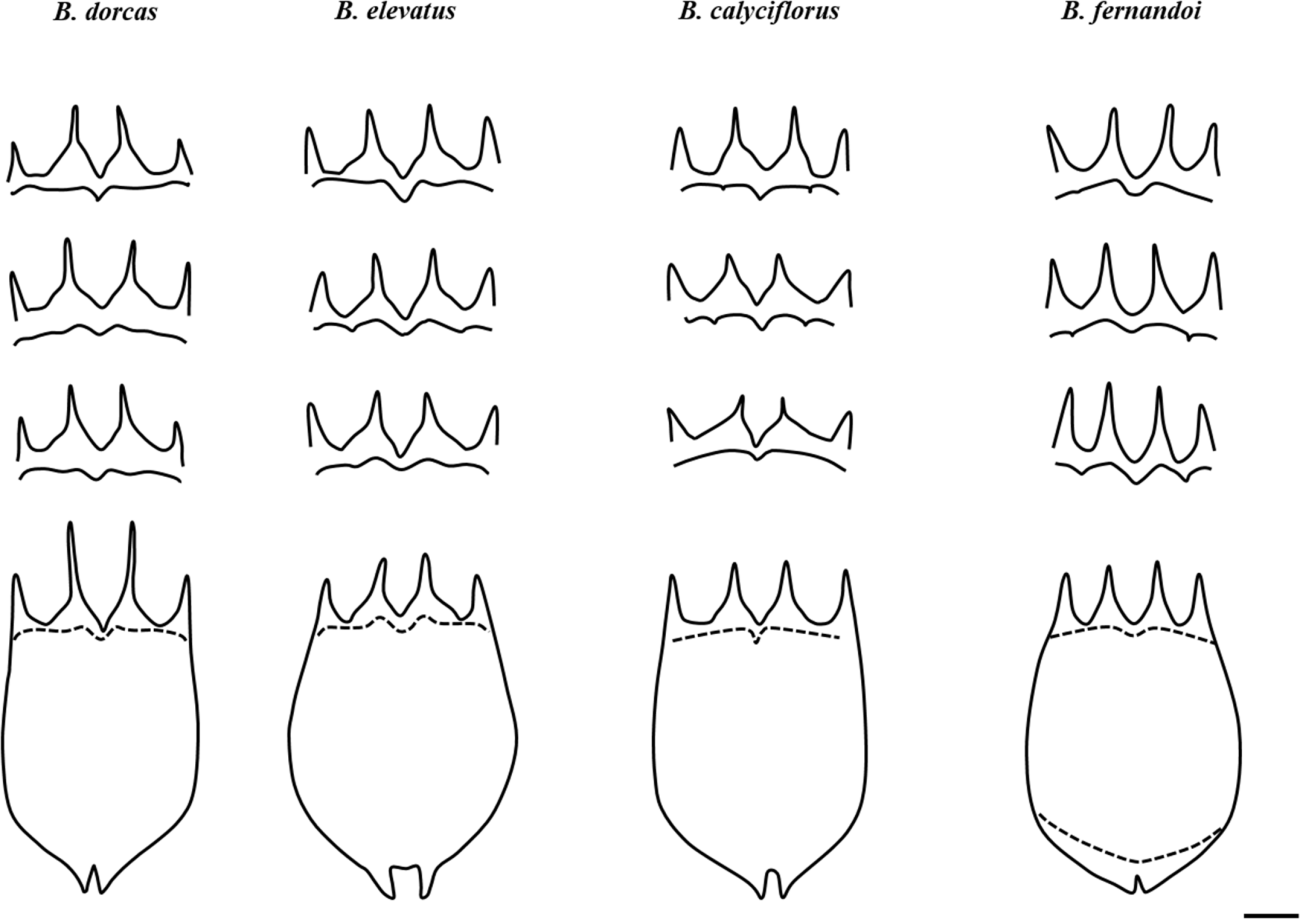
Line drawings of the main variations of the anterodorsal and anteroventral sides encountered for *B*. *dorcas*, *B*. *elevatus* sp. nov., *B*. *calyciflorus* s.s. and *B*. *fernandoi* sp. nov.

The original description of *B*. *calyciflorus* [58] lacks details. More recent authors [38, 30] describing a typical form of *B*. *calyciflorus* indicated a great variation in the morphology of the antero-dorsal spines. Of all the material examined in the present study and Papakostas et al. [20], species ‘C’ also exhibited the greatest variation in the morphology of the antero-dorsal spines (Fig 4) and was also characterized by the undulated anteroventral margin that is also mentioned by Koste [38] and Kutikova and Fernando [39]. Thus, species ‘C’ was identified as *B*. *calyciflorus*.

**Fig 5:**
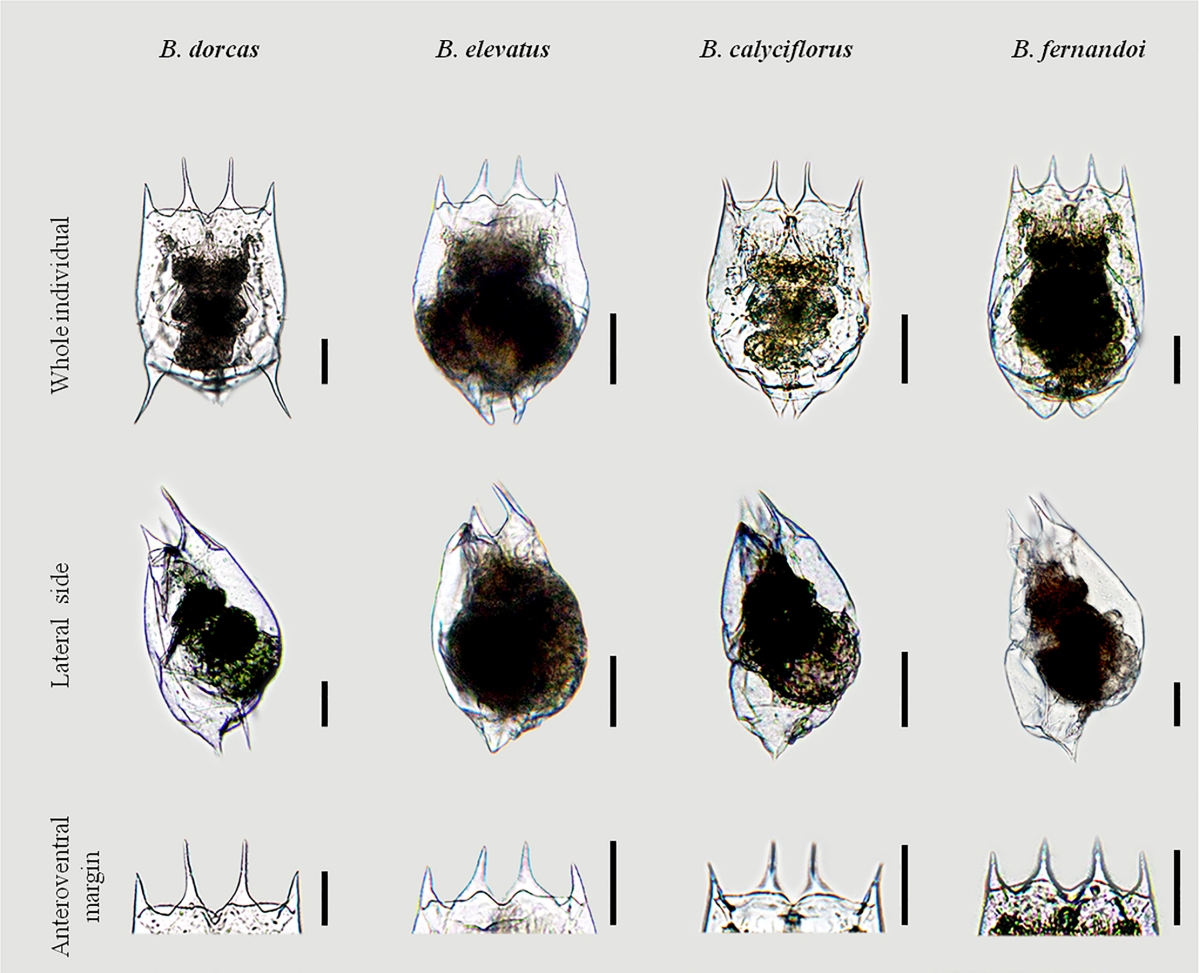
Photomicrographs of *B*. *calyciflorus* s.s., *B*. *dorcas*, *B*. *elevatus* sp. nov., *B*. *fernandoi* sp. nov. Whole individual, Lateral side, Anteroventral margin.

**Fig 6:**
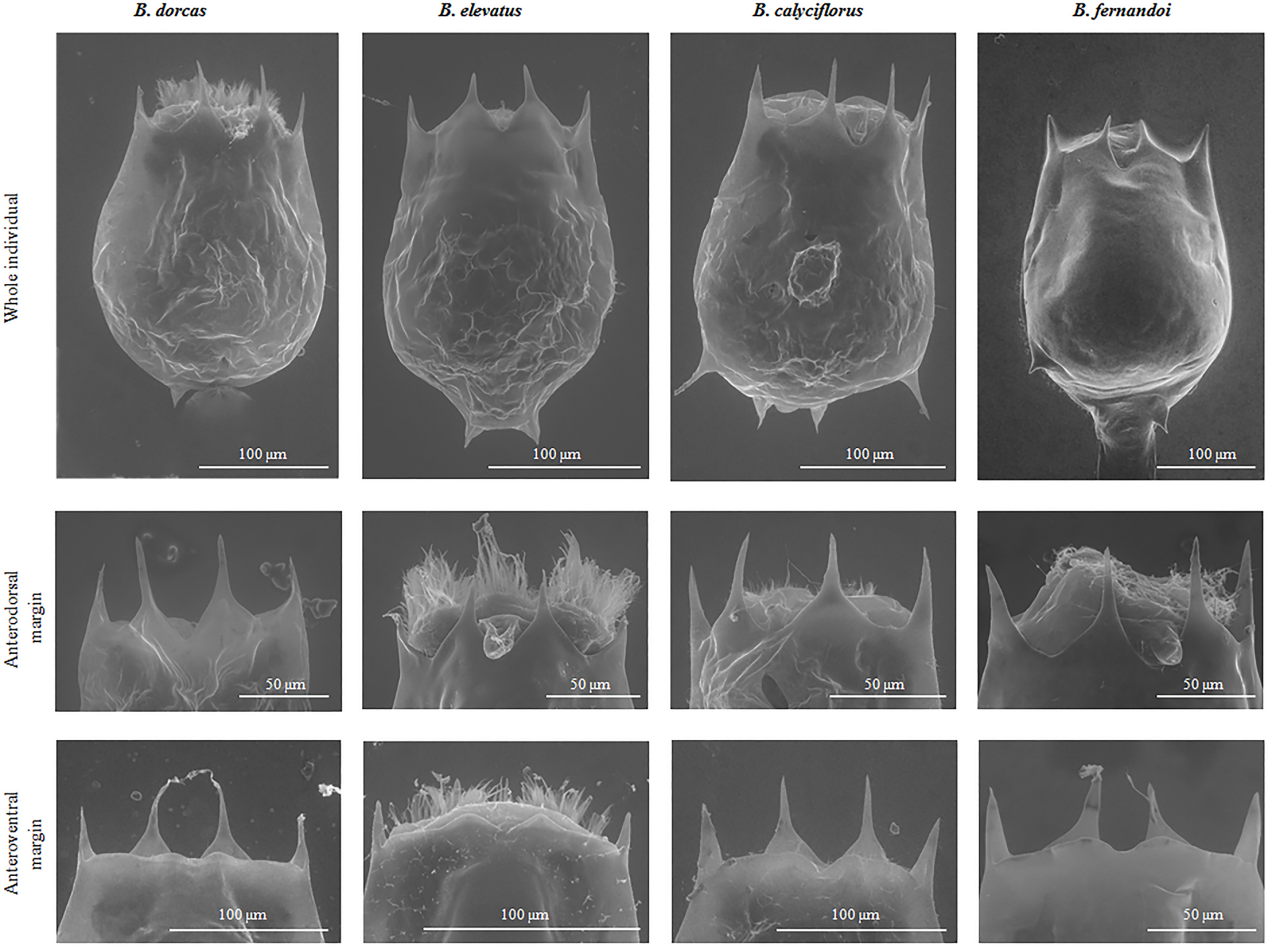
Scanning electron micrographs of *B*. *calyciflorus* s.s., *B*. *dorcas*, *B*. *elevatus* sp. nov., *B*. *fernandoi* sp. nov. Whole individual, Anterodorsal margin, Anteroventral margin.

Parthenogenetic female: Lorica saccate soft and ovoid- shaped with a smooth surface (Figs 4–6). Anterior dorsal margin with four spines, two on each side of a U-shaped sinus (Figs 4–6). All spines are triangular with a wide base and relatively sharp apices. The anterior ventral margin is smooth with a medial sinus (Figs 4–6). Foot aperture sub-terminal on the ventral surface of the lorica between two triangular protrusions. Three antennae are present: one found in the U-shaped sinus between the medial anterodorsal spines when the coronal disc is extended, and two others on either lateral side of the lorica at the posterior part of the animal length.

**Fig 7:**
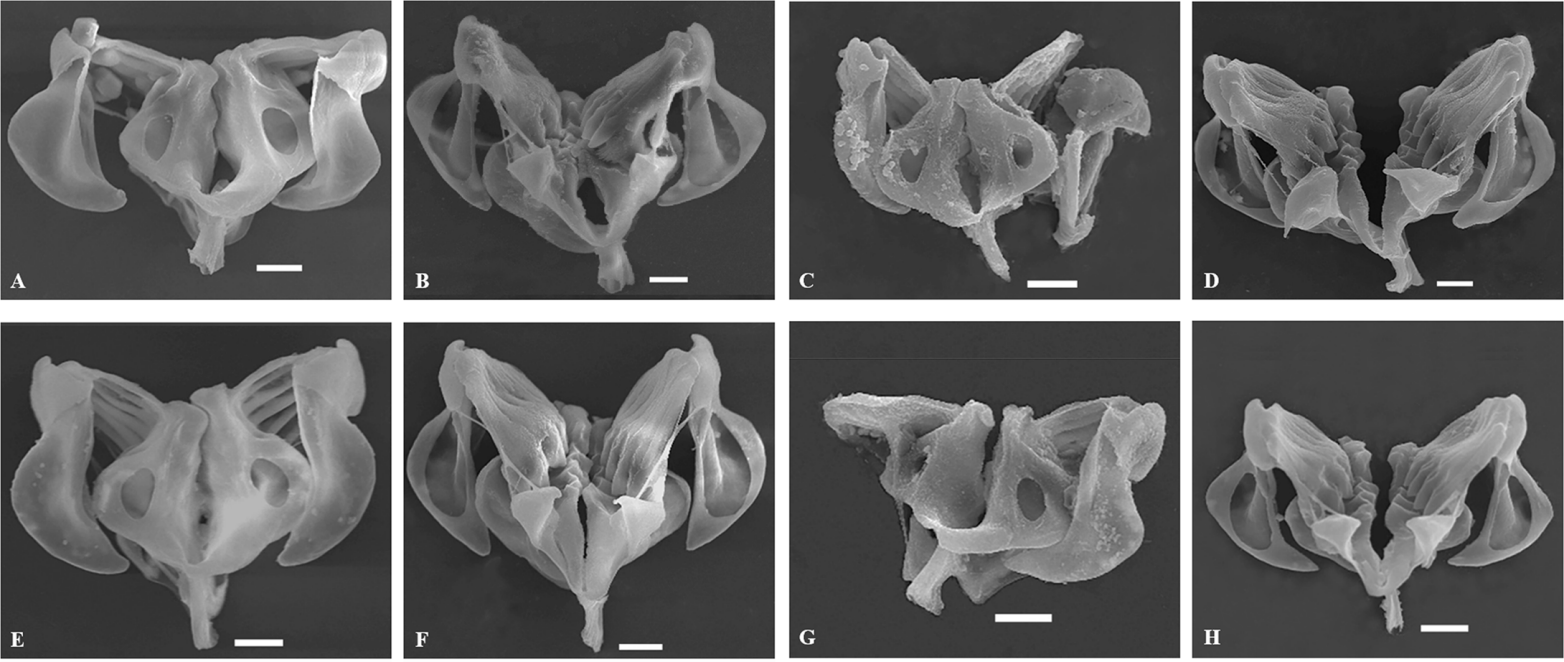
Scanning electron micrographs of the trophi dorsal and ventral view for *B*. *dorcas* (A, B), *B*. *elevatus* sp. nov. (C, D), *B*. *calyciflorus* s.s. (E, F) and *B*. *fernandoi* sp. nov. (G, H).

Trophi: Malleate type (Fig 7) bearing the characteristics of the genus as described by Segers et al. [69]. Fulcrum short and hollow. Rami asymmetrical. Unci with four or five teeth decreasing in size from the ventral one. Subuncus brush-like. Manubria are triangular in shape, rounded at the external sickle-shaped margin, flattened and slightly bend at their distal end.

#### Comments

Kutikova and Fernando [39] in their analysis of the *Brachionus calyciflorus* Pallas, 1766 variations referred to the typical form as the ‘d’ form. Their depiction of the anteroventral side is not of great accuracy, although the undulated anteroventral margin with only a median notch is depicted. As for the anterodorsal side, the same extent of variation can be seen as the one described in the present study. This can be identified with species ‘C’ as suggested by the molecular analysis of this study and of Papakostas et al. [20]. A genotype of *B*. *calyciflorus* s.s. was used as material for the whole genome sequencing by [36].

#### Distribution-Habitat

*Brachionus calyciflorus* s.s. has a cosmopolitan distribution. Based on the material analysed the present study confirms morphologically and genetically its Palearctic distribution. Relating our species with the typical form of Kutikova & Fernando [39] its Tropical, Oriental and Australian distribution is also confirmed, while it also has a Nearctic distribution (Table A in S1 Text).

It is an euplanctonic species found in freshwater pools, ponds, lakes, and reservoirs, ditches and paddy fields; also in potamoplankton, river estuaries, coastal brackish and marine waters; prefers eu- to hypertrophic waters, circum-neutral to slightly alkaline conditions, tolerates low oxygen [51].

### Redescription of *Brachionus dorcas* Gosse, 1851

#### Taxonomy

Class: Eurotatoria De Ridder, 1957
Subclass: Monogononta Plate, 1889
Superorder: Pseudotrocha Kutikova, 1970
Order: Ploima Hudson & Gosse, 1886
Family: Brachionidae Ehrenberg, 1838
*Brachionus dorcas* Gosse, 1851

*Brachionus dorcas* Gosse 1851 [70], p. 203,

*Brachionus calyciflorus dorcas*, Gosse 1851 [70], p. 203

*Brachionus dorcas spinosus*, Wierzejski 1891 [71], p. 52, Fig 4

#### Etymology

The name ‘dorcas’ comes from the Greek word ‘δορκάς’ which is an antelope. Its use refers to the middle anterodorsal spines which resembled the horns of an antelope according to the description made by Gosse [70] ‘*Lorica ovate or subconical; occipital edge with four long slender spines*, *the middle pair curving forwards*, *and bent first from*, *and then towards*, *each other*, *like horns of an antelope; mental edge undulated*, *with a notch in the centre’*

#### Material examined

A total of 151 individuals were examined coming from 11 clones established from resting eggs collected in 5 water bodies in The Netherlands (Table 1).

Permanent glycerin glass slide mounts (voucher specimens), each containing a single specimen, were prepared according to Jersabek et al. [68], and deposited in the Frank J. Myers collection at the Academy of Natural Sciences in Philadelphia (ANSP) with catalogue numbers ANSP [2105–2110].

Based on the literature review (S1 Text) and the information available in the LAN [9, 50] we conclude that no type material of *B*. *calyciflorus* is available. Following the guidelines of ICZN, since we are dealing with a species complex, we decided to designate a specific slide as neotype.

Neotype: A parthenogenetic female in a permanent glycerin glass with catalogue number [2105]

#### Description

Of all the examined material Clones 7J and C02NL134 were identified as the *B*. *dorcas* Gosse, 1851 first described by Gosse [70] due to its biggest median anterodorsal spines; thus the following description is based on those individuals.

Parthenogenetic female: Lorica saccate soft and ovoid- shaped with smooth surface (Figs 4–6). Anterior dorsal margin with four spines, two on each side of a V-shaped sinus (Figs 4–6). All spines are triangular with a wide base and relatively sharp apices. Median spines are longer compared to the lateral spines. The anterior ventral margin has a wave-like shape on each side of a medial sinus (Figs 4–6). No lateral notches exist. Foot aperture sub-terminal on the ventral surface of the lorica between two triangular protrusions. Three antennae are present: one found in the V-shaped sinus between the medial anterodorsal spines when the coronal disc is extended, and two others on either lateral side of the lorica slightly posterior to the midpoint of the animal length.

Trophi: Malleate type (Fig 7) bearing the characteristics of the genus as described by Segers et al. [69]. Fulcrum short and hollow. Rami asymmetrical. Unci with four or five teeth decreasing in size from the ventral one. Subuncus brush-like. Manubria are triangular, pointed at the external sickle-shaped margin, flattened and bend at their distal end.

#### Comments

The discriminating factor of the individuals of *Brachionus dorcas* is related to the anterodorsal spines, and specifically the fact that the median spines were much longer compared to the other groups. This character is among the ones used by Koste [38] to describe the *Brachionus calyciflorus* variation *dorcas*. Nevertheless, *Brachionus dorcas* was initially described by Gosse [70] at the species level. Its description can be identified as a match with species ‘A’ of the present study and the study of Papakostas et al. [20]. The results of the present analysis along with the analysis presented by Papakostas et al. [20] justify the establishment of *Brachionus dorcas* as a valid species.

#### Distribution-Habitat

*Brachionus dorcas* has a Palearctic distribution confirmed genetically and morphologically by the material analysed the present study. It is also known to have a Tropical, Oriental and Australian distribution (Table A in S1 Text).

It is an euplanktonic species found in freshwater pools, ponds, tanks, lakes and reservoirs, ditches and paddy fields; also in potamoplankton, river estuaries, coastal, brackish and marine waters; prefers eu- to hypertrophic waters, circum-neutral to slightly alkaline conditions, tolerates low oxygen; eurytherm but prefers warm waters (Table A in S1 Text).

### Description of *Brachionus elevatus* sp.nov

urn:lsid:zoobank.org:act:624FD96E-9566-4D38-A5CB-86F75649B349

#### Taxonomy

Class: Eurotatoria De Ridder, 1957
Subclass: Monogononta Plate, 1889
Superorder: Pseudotrocha Kutikova, 1970
Order: Ploima Hudson & Gosse, 1886
Family: Brachionidae Ehrenberg, 1838
Brachionus elevatus

#### Etymology

The name *elevatus* refers to the elevation of the anteroventral margin right before the median notch. It comes form the Latin elevatus, past participle of elevare "lift up, raise".

#### Type locality

Shallow eutrophic city pond close to city center of The Hague (The Netherlands); N 52.090694°; E 4.338444°

#### Material examined

A total of 254 individuals were examined coming from 14 clones established from resting eggs collected in 3 water bodies from The Netherlands (Table 1).

Of all the clones examined clones 69H and C04NL7 has been chosen to formally describe *Brachionus elevatus* sp. nov. Permanent glycerin glass slide mounts, each containing a single specimen, were prepared according to Jersabek et al. [68], and deposited in the Frank J. Myers collection at the Academy of Natural Sciences in Philadelphia (ANSP).

Holotype: A parthenogenetic female in a permanent glycerin glass slide with catalogue number ANSP [2111].

Paratypes: A total of 9 slides with catalogue number ANSP [2112–2120].

#### Description

Parthenogenetic female: Lorica saccate soft and ovoid- shaped with a smooth surface (Figs 4–6). Anterior dorsal margin with four spines, two on each side of a V-shaped sinus (Figs 4–6). All spines are triangular with a wide base and relatively sharp apices. The anterior ventral margin has a wave-like shape on each side of a medial sinus (Figs 4–6). The medial sinus is elevated with a well-marked median notch between short oval or nearly triangular protuberances. No lateral notches exist. Foot aperture sub-terminal on the ventral surface of the lorica between two triangular protrusions. Three antennae are present: one found in the V-shaped sinus between the medial anterodorsal spines when the coronal disc is extended, and two others on either lateral side of the lorica slightly posterior to the widest point of the lorica.

Trophi: Malleate type (Fig 7) bearing the characteristics of the genus as described by Segers et al. [69]. Fulcrum short and hollow. Rami asymmetrical. Unci with four or five teeth decreasing in size from the ventral one. Subuncus brush-like. Manubria are more similar in shape with the ones of *B*. *calyciflorus* with rounded external margin flattened and slightly bend at their distal end.

#### Comments

In the present study, the anteroventral structure with the marked protuberances of the medial sinus discriminated the individuals of the *Brachionus elevatus* from the individuals of the *B*. *calyciflorus* s.s. Kutikova & Fernando [39] based on this characteristic describe their ‘b’ form as being intermediate between *Brachionus calyciflorus borgerti* Apstein, 1907 and the typical form in the sense that the ‘b’ form lacks the saw-like basal tooth in the median spines of the anterodorsal margin of *Brachionus calyciflorus borgerti* although they have a broad base. They also hypothesize that this intermediate form might be a hybrid. Based on the analysis of Papakostas et al. [20] and the present study, this hypothesis can be rejected.

#### Distribution-Habitat

*Brachionus elevatus* sp. nov. has a Palearctic distribution confirmed genetically and morphologically by the material analysed in the present study. Relating the species described in the present study with the ‘b’ form described by Kutikova & Fernando [39] *B*. *elevatus* sp. nov. can be considered to have an Oriental distribution as well. It is found in freshwater habitats and it is an euplanktonic species.

### Description of *Brachionus fernandoi* sp.nov

urn:lsid:zoobank.org:act:0A4F5205-47ED-47ED-4C72-9686-0E33E0F34DA3

#### Taxonomy

Class: Eurotatoria De Ridder, 1957
Subclass: Monogononta Plate, 1889
Superorder: Pseudotrocha Kutikova, 1970
Order: Ploima Hudson & Gosse, 1886
Family: Brachionidae Ehrenberg, 1838
Brachionus fernandoi

#### Etymology

The species is named after dr. C. H. Fernando for his contribution to the description of the *Brachionus calyciflorus* variations in the paper Kutikova & Fernando [39].

Type locality

Eutrophic ditch within residential area, near the town of Hellevoetsluis (The Netherlands); N 51.839032°; E 4.144425°

#### Material examined

A total of 136 individuals were examined coming from 10 clones established from resting eggs collected in 3 water bodies from the Netherlands (Table 1).

Of all the clones examined C02NL181 has been chosen to formally describe *Brachionus fernandoi* sp. nov. Permanent glycerin glass slide mounts, each containing a single specimen, were prepared according to Jersabek et al. [68], and deposited in the Frank J. Myers collection at the Academy of Natural Sciences in Philadelphia (ANSP).

Holotype: A parthenogenetic female in a permanent glycerin glass slide with catalogue number ANSP [2121].

Paratypes: A total of 4 slides with catalogue number ANSP [2122–2125].

#### Description

Parthenogenetic female: Lorica saccate soft and ovoid- shaped with a smooth surface (Figs 4–6). Anterior dorsal margin with four spines, two on each side of a U-shaped sinus (Figs 4–6). All spines are triangular with a wide base and relatively sharp apices. The anterior ventral margin is smooth with a wide medial sinus (Figs 4–6). Foot aperture sub-terminal on the ventral surface of the lorica between two triangular protrusions of varying length. This posterior ventral part is swollen. Three antennae are present: one found in the U-shaped sinus between the medial anterodorsal spines when the coronal disc is extended, and two others on either lateral side of the lorica slightly posterior to the midpoint of the lorica’s length.

Trophi: Malleate type (Fig 7) bearing the characteristics of the genus as described by Segers et al. [69]. Fulcrum short and hollow. Rami asymmetrical. Unci with four or five teeth decreasing in size from the ventral one. Subuncus brush-like. Manubria more similar in shape with *B*. *dorcas*, are triangular, pointed at the external sickle-shaped margin, flattened and bend at their distal end.

#### Comments

Based on the forms described by Kutikova & Fernando [39] *Brachionus fernandoi* sp. nov. seems to resemble the ‘c’ form they describe. They mention ‘*a very swollen posterior part of the dorsal plate*’. In our opinion, the posterior ventral part is swollen giving the impression of the swollen dorsal part. This can be identified as a match with species ‘D’ of the present study and the study of Papakostas et al. [20].

#### Distribution-Habitat

*Brachionus fernandoi* sp. nov. has a Palearctic distribution confirmed genetically and morphologically by the material analysed in the present study. Relating the species described in the present study with the ‘c’ form described by Kutikova & Fernando [39] then *B*. *fernandoi* sp. nov. can be considered to have an Oriental distribution as well. It is found in freshwater habitats, and it is an euplanktonic species.

### Differential diagnosis

The morphology of the anteroventral margin appears to be a powerful trait. This margin is characterized by a wave-like shape in *B*. *dorcas* and *B*. *elevatus* sp. nov. whereas in *B*. *calyciflorus* s.s. and *B*. *fernandoi* sp. nov. it is mainly completely smooth. This latter character though exhibits a lot of variation (Fig 5).

*Brachionus dorcas* is discriminated from the other three species based on the size of the anterodorsal spines. *B*. *dorcas* has much longer median anterodorsal spines compared to the other species. This was the case for all three dimensions measured concerning this characteristic; *B*. *dorcas*: e (range 43.84–92.62 μm), k (range 52.41–86.21 μm) and j (range 46.32–98.38 μm), *B*. *calyciflorus* s.s. e (range 25.17–69.82 μm), k (range 31.14–68.89 μm) and j (range 25.15–74.05 μm), *B*. *elevatus* sp. nov. e (range 29.54–74.2 μm), k (range 32.77–73.83 μm) and j (range 33.17–75.56 μm). *B*. *fernandoi* sp. nov. e (range 34.62–72.31 μm), k (range 40.77–70.96 μm) and j (range 38.07–73.77 μm) (S2 Table).

*B*. *elevatus* sp. nov. is characterized by the fact that the medial sinus is elevated with a well-marked median notch between short oval or nearly triangular protuberances. Further discrimination between *B*. *dorcas* and *B*. *elevatus* sp. nov. is based on a combination of traits. In the case of *Brachionus dorcas* the depth of the anterodorsal medial sinus ‘e’ is usually > 60 μm and the width of the anteroventral medial sinus is smaller than 1/5 of the distance of the lateral anterodorsal spines (i.e., r/b < 0.20). The dimensions measured concerning these characteristics for *B*. *dorcas* and *B*. *elevatus* sp. nov. are: *B*. *dorcas* e (range 43.84–92.62 μm, mean 67.16), r/b (range 0.102–0.253, mean 0.187), *B*. *elevatus* sp. nov. e (range 29.54–74.2 μm, mean 49.82), r/b (range 0.164–0.424, mean 0.254) (S2 Table).

*Brachionus fernandoi* sp. nov. compared to *B*. *elevatus* sp. nov. has no protrusion right before the median sinus of the anteroventral margin which is smooth with no lobes being formed by intermediate notches. The morphological trait that discriminates *B*. *fernandoi* sp. nov. from *B*. *calyciflorus* s.s. is the opening of the median sinus of the anterovental margin which has the biggest opening; r range 21.33–45.50 *B*. *fernandoi* sp. nov., 14.75–40.66 *B*. *dorcas*, 13.47–38.25 *B*. *calyciflorus*, 15.95–49.17 *B*. *elevatus* sp. nov. Additionally, *B*. *fernandoi* sp. nov. has a narrower anteroventral opening ‘i’, while *B*. *calyciflorus* s.s. has a wider anterodorsal side ‘b’.

#### Key to the identification of the four species of the *Brachionus calyciflorus* species complex

- Wave-like anteroventral margin……………………………………………………2
  - Anterovental margin either smooth or with notches ……………….……………3
- Median anterodorsal spines longer than lateral anterodorsal spines …………………………………………………………………………*Brachionus dorcas*
  - Anterodorsal spines of more or less equal length; anteroventral margin with an elevation right before the medial sinus ………………*Brachionus elevatus* sp. nov
- Width of the anteroventral medial sinus > 30 μm and more than 1/5 the lorica width (i.e. r/c ratio > 0.2) ……………………………………………*Brachionus fernandoi* sp. nov.
  - Width of the anteroventral medial sinus <30 μm and less than 1/5 the lorica width (i.e. r/c ratio < 0.2) …………………………………………….*Brachionus calyciflorus* s.s.

Based on the above key as well as the discriminating characters we performed a blind test on 20 individuals randomly selected from the 724 individuals analysed. Only two cases were misidentified. This 10% of misidentified cases is close to the results of the DFA where in total 91% of the cases where correctly classified.

## Discussion

By applying the approach of reverse taxonomy [14], we confirmed the existence of four putative species as predicted by Papakostas et al. [20] based on molecular species delimitation techniques. Indeed, a morphometric analysis of 724 individuals from 48 different clones originating from 10 Dutch water bodies, revealed a clear differentiation among each of the four species pairs. Also, by combining a morphological analysis with an exhaustive literature survey, we were able to link the four species to previously described forms.

Our study highlights specific morphological traits that were found to be particularly useful for the distinction between species of the *B*. *calyciflorus* species complex. This was especially the case for features of the anteroventral side. Such features have long been suspected a strong diagnostic character for the genus *Brachionus* [37]. Our results thus confirmed the notion by Kutikova and Fernando [39] that aspects of both the anterodorsal and anteroventral side may be useful diagnostic characters for differentiating among *B*. *calyciflorus* forms. Similarly, these traits were proven valuable for the discrimination between *B*. *asplanchnoidis* Charin, 1947 and other members of the *B*. *plicatilis* species complex [26].

Conversely, the presence and size of posterolateral spines proved of little taxonomic value. Historically, these traits have been used to describe certain forms of *Brachionus calyciflorus* (i.e., *B*. *calyciflorus* f. *anuraeiformis* and *B*. *calyciflorus* f. *amphiceros* [38, 60, 72]). From our analysis, it was evident that lateral spines cannot be used as a taxonomic character because the frequency of occurrence (ranging within clones between 0 to 100%) as well as the length of these spines varied strongly among clones that belonged to the same species or even among individuals from the same clone. Besides, it is well documented that the lateral spines are the result of exogenous factors, such as predation or food concentration [73, 74, 75, 76]. Thus, the morphological forms that have been previously assigned based on the presence of lateral spines have no taxonomic validity [9].

The validity and applicability of our suggested diagnostic traits still need verification using field samples. Given that the prime objective of this study was to test the hypothesis of four morphospecies as suggested by molecular methods, we excluded as much as possible phenotypic variation associated to environmental variability by culturing the investigated individuals under standardized laboratory conditions. Whereas this may have strongly increased our ability to differentiate among species it precludes variation resulting from phenotypic plasticity in response to environmental variability under natural conditions. We thus recognize that discrimination of individuals collected from the field may prove less straightforward than suggested by our analyses [26]. For example, in the case of *B*. *plicatilis* Müller, 1786, morphological identifications based upon individuals raised under laboratory conditions [24] have proven to be inadequate for field studies in some cases [17, 77]. Although our diagnostic traits seemed to provide good resolution to distinguish between the four species of the *B*. *calycilforus* complex, we anticipate that future research will clarify the extent to which this may be true for field samples.

Another point of consideration when interpreting our conclusions is related to the restricted geographic distribution of our studied samples. In our experimental design, we tried to incorporate interpopulation genetic variation by investigating multiple clones from different populations per species. Such approach prevents that the results of morphometric analyses are contingent on particular genotypes or populations and as such guarantees robustness and generality of reported differences among species. However, all investigated populations originated from a relatively restricted geographic area (i.e., the territory of The Netherlands). Consequently, the morphological and morphometric variation reported in this study may constitute an underrepresentation of what exists for each of the species throughout their biogeographic range. Although this may limit the generality of the diagnostic traits suggested for species identification, this does not disqualify our conclusion that systematic differences between species exist.

Future work should also clarify how hybrid introgression among the species of the *B*. *calyciflorus* complex impacts morphology. Papakostas et al. [20] provided strong evidence for hybrid formation and introgression among the species of the *B*. *calyciflorus* complex. However, this has not prevented us from finding clear morphometric differences between the species. Kutikova and Fernando [39] described forms of *B*. *calyciflorus* that correspond to some of the species described in this work. They also reported the existence of cases with intermediate features and hypothezised them to be the result of hybridization. Investigating how gene flow among species would affect morphometric traits may thus represent an intriguing topic of research to be done.

## Supporting information

S1 Text Records of *Brachionus calyciflorus* synonyms. (DOCX)

S1 Table Amount of morphometric variation explained by species identity for each pair of the investigated species. (DOCX)

S2 Table Summary statistics for the selection of morphometric traits measured on individuals of the four species. (DOCX)

S3 Table Classification functions of the stepwise discriminant analysis performed on species ‘A’, ‘B’, ‘C’, and ‘D’.
(DOCX)

S4 Table Classification functions of the stepwise discriminant analysis performed on species ‘B’, ‘C’, and ‘D’.
(DOCX)
