# Supplementary material for: Reverse taxonomy applied to the Brachionus calyciflorus cryptic species complex: Morphometric analysis confirms species delimitations revealed by molecular phylogenetic analysis and allows the (re)description of four species
Source: PLoS One. 2018 Sep 20;13(9):e0203168. doi: 10.1371/journal.pone.0203168 (PMC6147415; doi:10.1371/journal.pone.0203168)
Supplement: S1 Text — (DOCX) [file pone.0203168.s001.docx]

**Reverse taxonomy applied to the *Brachionus calyciflorus* cryptic species complex: morphometric analysis confirms species delimitations revealed by molecular phylogenetic analysis and allows the (re)description of four species.**

Evangelia Michaloudi^1^*, Spiros Papakostas^2^, Georgia Stamou^1^, Vilém Neděla^3^; Eva Tihlaříková^3^; Wei Zhang^4^; Steven A.J Declerck^4^

^1^ Department of Zoology, School of Biology, Aristotle University of Thessaloniki, Τhessaloniki, Greece

^2^ Division of Genetics and Physiology, Department of Biology, University of Turku, Turku, Finland

^3^ Institute of Scientific Instruments, Academy Of Sciences of the Czech Republic, Brno, Czech Republic

^4^ Netherlands Institute of Ecology, Department of Aquatic Ecology, Wageningen, The Netherland

* Corresponding author: E-mail: tholi@bio.auth.gr (EM)

**Supporting Information**

Table A. Records of *Brachionus calyciflorus* synonyms with notes on the name used

| **Country** | **Lake, River or Area** | **Source reference** | **Name** |
| --- | --- | --- | --- |
|  | Siberia | Ehrenberg (1830) | *Anuraea palea* |
|  |  |  | *B. palea* |
| Germany | Berlin | Ehrenberg (1838) | *B. amphiceros* |
| Russia | Saint Petersburg | Weisse (1845) | *Anuraea divaricata* |
| United Kingdom | Britain | Gosse (1851) | *B. dorcas* |
|  |  |  | *B. amphiceros* |
|  |  |  | *B. pala* |
| Egypt | Cairo | Schmarda (1854) | *Arthracanthus biremis* |
|  | Monfalut |  | *Arthracanthus quadriremis* |
|  | Assiut |  |  |
|  | Sakura |  |  |
|  | Medinet Habu |  | *B. pala* |
| - |  | Cohn (1862) | *B. amphiceros* |
| Hungary | Mezoseg | Daday (1883) | *B. margoi* |
| United Kingdom | Walthamstow | Hudson & Gosse (1886) | *B. dorcas* |
|  | Ponds and ditches |  | *B. amphiceros* |
| Germany | Bonn | Plate (1886) | *B. amphiceros* |
|  |  |  | *B. decipiens* |
| Austria-Hungary | Galicie | Wierzejski (1891) | *B. dorcas* |
|  |  |  | *B. dorcas* var. *spinosus* |
| Hungary |  | France (1894) | *B. pentacanthus* |
| Ukraine | Kharkov | Skorikov (1896) | *B. spinosus* |
|  |  |  | *B. pala* |
|  |  |  | *B. dorcas* |
| Switzerland | Jonction | Weber (1898) | *B. pala* |
|  |  |  | *B. pala* var. *amphiceros* |
| Germany |  | Zacharias (1898) | *B. amphiceros* |
|  |  |  | *B. amphiceros* var*. pala* |
| Romania | Mezö-Zäh | Daday (1901) | *B. pala* var. *amphiceros* |
|  |  |  | *B. dorcas* |
| Russia | Vyatka River | Zernov (1901) | *B. amphiceros* |
|  |  |  | *B. pala* |
| Russia | Volga River | Meissner (1902) | *B. pala* |
| Scotland |  | Murray (1906) | *B. pala* |
| South Africa |  | Rousselet (1906) | *B. pala* var. *dorcas* |
|  |  |  | *B. pala* |
| Sri Lanka | Colombo Lake | Apstein (1907) | *B. amphiceras* var. *borgerti* |
|  |  |  | *Brachionus pala* |
| Shanghai |  | Brehm (1909) | *B. pala* var. *anuraeiformis* |
|  |  |  | *B. pala* |
| Germany |  | Collin et al. (1912) | *B. pala* |
|  |  |  | *B. pala* f. *amphiceros* |
|  |  |  | *B. pala* var. *dorcas* |
|  |  |  | *B. pala* var. *dorcas* f*. spinosa* |
| Germany | Wallgraben | Dieffenbach & Sachse (1912) | *B. dorcas* |
|  |  |  | *B. dorcas* var. *spinosus* |
|  |  |  | *B. amphiceros* |
|  |  |  | *B. pala* |
| United States | Winona Lake | Henry (1913) | *B. pala* |
| Swede n | Stockholm | Huss (1913) | *B. pala* |
| Germany | Mansfelder | Colditz (1914) | *B. pala* |
| Romania | Bukowina | Hartmann (1915) | *B. pala* f. *amphiceros* |
|  |  |  | *B. pala* var. *dorcas* |
|  |  |  | *B. pala* var. *dorcas* f*. spinosa* |
| Spain | Albufera de Valencia | Arévalo (1918a) | *B. pala* f. *amphiceros* |
|  |  |  | *B. pala* var. *dorcas* f*. spinosa* |
|  |  |  | *B. pala* |
|  | Barcelona | Arévalo (1918b) | *B. pala* |
| Netherlands | Zuidlaardermeer | Havinga (1919) | *B. pala* |
|  |  |  | *B. pala* f*. amphiceros* |
| United States | Salton Sea | Allen (1920) | *B. pala* |
| Switzerland | Egelmösli | Schreyer (1920) | *B. dorcas* |
|  |  |  | *B. dorcas* var. *spinosa* |
|  |  |  | *B. pala* |
|  |  |  | *B. pala* var. *anuraeiformis* |
| Czech Republic | Brno | Spandl (1922) | *B. pala* var*. mucronatus* |
| Armenia |  | Spandl (1923) | *B. pala* var. *dorcas* |
| United States | Java Lake | Van Oye (1954) | *B. pala* |
| Russia | Pe tschora-Beckens | Decksbach (1926) | *B. pala* |
| Germany | Seeburger See | Künne (1926) | *B. pala* |
|  |  |  | *B. pala* f*. amphiceros* |
|  |  |  | *B. pala* var. *dorcas* |
|  |  |  | *B. pala* var. *dorcas* f*. spinosus* |
| Finland |  | Välikangas (1926) | *B. pala* |
| Russia | Pond near Leningrad | Rylov (1927) | *B. pala* var. *amphiceros* |
| State of Palestine |  | Krampner (1928) | *B. pala* |
| Germany | Greifswald | Stammer (1928) | *B. pala* |
| Czech Republic | Žákovice | Watzka (1928) | *B. pala* |
| Ethiopia | Abyssinian Fresh Waters | Bryce (1931) | *B. calyciflorus* *amphiceros* |
| Kenya | Lac Naivasha | De Beauchamp (1932a) | *B. pala* f. *amphiceros* |
|  |  |  | *B. pala* |
|  | Lake Rudolf | De Beauchamp (1932b) | *B. pala* |
|  | Lac Naivasha |  |  |
| Uganda | Lake Nakavali |  |  |
|  | Lake Kijanebalola |  |  |
|  | Lake Edward |  |  |
|  |  |  | *B. pala* var*. amphiceros* |
|  | Lake George |  |  |
|  |  |  | *B. pala* var. *spinosus* |
| Switzerland | Luzern | Steinmann & Surbeck (1932). | *B. pala* |
| Germany | Chiemgau | Aurich (1933) | *B. pala* |
|  |  |  | *B. pala* f. *amphiceros* |
|  |  |  | *B. pala* f. *anuraeiformis* |
| United States | St. Joseph River | Dolley (1933) | *B. pala* |
| Taiwan | Formosa |  | *B. calyciflorus* f. *borgerti* |
| Germamy | Ruhr | Lehmann & Westf (1933) | *B. pala* |
| Netherlands | Zuiderzee | Redeke (1935) | *B. pala* |
| India |  | Seymour-Sewell (1935) | *B. pala* var*. dorcas* |
|  |  |  | *B. amphiceras* |
| Germany | Solln Lake | Buchner (1937) | *B. pala* |
|  | Taufkirchen Lake |  |  |
|  | Etterschlag Lake |  |  |
|  | Teltow |  |  |
| Belgium | Meuse River | Damas (1939) | *B. pala* |
| Peru | Lac Titicaca | De Beauchamp (1939) | *B. pala* |
|  | River Capachica |  |  |
| Turkey | Belgratwald | Mann (1940) | *B. pala* f. *dorcas* |
| Belgium | Meuse River | Damas (1939) | *B. pala* |
| France | Rambouillet | Lefèvre (1941) | *B. pala* |
| Philippines | Lake Taal | Woltereck et al. (1941) | *B. calyciflorus dorcas* |
|  |  |  | *B. calyciflorus dorcas spinosa* |
| United States | Green Pond | Myers (1942) | *B. calyciflorus* f. *amphiceros* |
| Czech Republic | Chotěboř pond | Bartoš (1948) | *B. calyciflorus* f. *dorcas* |
| India | Bankipore | Donner (1949) | *B. pala* |
| United States | Oklahoma pond | Brown & McDaniel (1952) | *B. pala* |
| England | Cambridgeshire | Gray (1953) | *B. pala* |
| Italia | Lentini | Bērzins (1954) | *B. calyciflorus calyciflorus* |
| - |  | Otto (1954) | *B. pala* |
|  |  |  | *B. pala* var*. amphiceros* |
| Czechoslovakian | Pond Heřmansky | Sládeček (1955) | *B. calyciflorus* f. *dorcas* |
| France | Rhône | Aguesse (1957) | *B. pala* |
| France | Etang de la Plaine | Pourriot (1957) | *B. pala* f. *dorcas* |
| Switzerland | Widen | Nipkow (1958) | *B. calyciflorus* f. *amphiceros* |
| Austria | Jaidhof-Teiche | Wawrik (1960) | *B. calyciflorus* var. *anuraeiformis* |
|  |  |  | *B. calyciflorus* f*. spinosa* |
| Germany | Munich | Buchner & Mulzer (1961) | *B. pala* |
|  |  |  | *B. calyciflorus* var. *dorcas* |
|  |  |  | *B. calyciflorus* var. *dorcas* f. *spinosa* |
| Japan | Japanese Inland Waters | Yamamoto (1960) | *B. calyciflorus dorcas* type |
|  |  |  | *B. calyciflorus* *anuraeiformis* type |
|  |  |  | *B. calyciflorus* *amphiceros* type |
|  |  |  | *B. calyciflorus* *dorcas spinosus* type |
| Iran | Iranian inland waters | Löffler (1961) | *B. calyciflorus* var. *dorcas* |
|  |  |  | *B. calyciflorus* f. *amphiceros* |
| South Africa | Zeekoe Vlei | Harisson (1962) | *B. calyciflorus* var. *dorcas* |
|  |  |  | *B. calyciflorus* var. *dorcas* f. *spinosa* |
|  |  |  | *B. calyciflorus* *amphiceros* |
| Sudan | Wadi Haifa | Löffler (1962) | *B. calyciflorus* var. *dorcas* |
| Japan |  | Sudzuki (1964) | *B. calyciflorus* var. *dorcus* |
|  |  |  | *B. calyciflorus* f. *anuraeiformis* |
|  |  |  | *B. calyciflorus* f. *amphiceros* |
| Brazil | Santarem | Hauer (1965) | *B. calyciflorus dorcas spinosa* |
|  | Lago Juanico |  | *B. calyciflorus* var. *mucronatus* |
| United States |  | Gilbert & Waage (1967) | *B. calyciflorus* var*. dorcas* |
|  |  |  | *B. calyciflorus* var*. pala* |
| Hungary | Danube | Kertész (1967) | *B. calyciflorus* var*. dorcas* |
|  |  |  | *B. calyciflorus* f. *anuraeiformis* |
|  |  |  | *B. calyciflorus* f. *amphiceros* |
|  |  |  | *B. calyciflorus calyciflorus* |
| Netherlands | Kievitsbuurt | Leentvaar (1967) | *B. calyciflorus* f. *pala* |
| Malaysia | Malacca | Dunn (1970) | *B. calyciflorus* var. *dorcas* f. *spinosa* |
| Chad | Lake Léré | Pourriot (1971) | *B. calyciflorus* f. *dorcas* |
| Chad | Madirom Channel | Robinson & (Robinson 1971) | *B. calyciflorus* *amphiceros* type |
| Austria | Waldviertels | Wawrik (1972) | *B. calyciflorus* var. *amphiceros* |
|  |  |  | *B. calyciflorus* var. *pala* |
| Netherlands | Volkerak-Haaringvliet | Peelen (1974) | *B. pala amphiceros* |
| Kenya | Lake Naivasha | Pejler (1974) | *B. calyciflorus* f. *pala* |
|  |  |  | *B. calyciflorus* f. *dorcas* |
|  |  |  | *B. calyciflorus* f. *spinosa* |
| Uganda | Lake Edwards |  | *B. calyciflorus* f. *pala* |
|  |  |  | *B. calyciflorus* f. *dorcas* |
|  |  |  | *B. calyciflorus* f. *spinosa* |
| Martinique | Martinigue Antilles | Pourriot (1975) | *B. calyciflorus* f. *dorcas* |
| Germany | Hase | Koste (1976) | *B. calyciflorus* f. *dorcas* |
|  |  |  | *B. calyciflorus* f. *amphiceros* |
|  |  | Koste (1978) | *B. calyciflorus* var. *dorcas* |
|  |  |  | *B. calyciflorus* f. *anuraeiformis* |
|  |  |  | *B. calyciflorus* f. *amphiceros* |
| Hungary | Tisza | Szabó & Keve (1977) | *B. calyciflorus* var. *dorcas* |
|  |  |  | *B. calyciflorus* var. *dorcas* f. *spinosa* |
| - | Caspian Sea | Marcuzzi (1979) | *B. pala* |
| India | Amtala | Sharma (1979) | *B. calyciflorus* var. *dorcas* |
|  | Budge Budge |  |  |
|  | Bon-Hoogly |  |  |
|  | Baripur |  |  |
|  | Sarisha |  |  |
|  | Shirakole |  |  |
|  | Tank Opposite Lindsay |  |  |
|  | Indian Museum Tank |  |  |
|  |  |  | *B. calyciflorus* f. *anuraeiformis* |
|  | Achipur |  |  |
|  | Manikpur |  |  |
|  | Calcuta |  | *B. calyciflorus* f. *borgerti* |
|  | Achipur |  |  |
|  | Amtala |  |  |
| Australia | Murray-Darling River | Koste & Shiel (1980) | *B. calyciflorus gigantea* |
| India | Orissa | Sharma (1980a) | *B. calyciflorus* var. *dorcas* |
|  |  |  | *B. calyciflorus* f. *anuraeiformis* |
| India | Panjab state | Sharma (1980b) | *B. calyciflorus* var. *dorcas* |
|  |  |  | *B. calyciflorus* f. *amphiceros* |
|  |  |  | *B. calyciflorus* f. *anuraeiformis* |
|  |  |  | *B. calyciflorus* f. *borgerti* |
| Australia | Murray-Darling system | Shiel (1980) | *B. calyciflorus calyciflorus* |
|  |  |  | *B. calyciflorus* var. *amphiceros* |
| Malaysia & Singapore | Malaysia & Singapore | Fernando & Zankai (1981) | *B. calyciflorus* f. *anuraeiformis* |
|  |  |  | *B. calyciflorus* f. *amphiceros* |
|  |  |  | *B. calyciflorus calyciflorus* |
| South African |  | Sartory (1981) | *B. calyciflorus* var. *dorcas* |
|  |  |  | *B. calyciflorus* var. *dorcas* f. *spinosa* |
|  |  |  | *B. calyciflorus* f. *amphiceros* |
| Romania | Lake Rosu | Godeanu & Zinevici (1983) | *B. calyciflorus dorcas* |
|  |  |  | *B. calyciflorus spinosa* |
|  | Lake Puiu |  | *B. calyciflorus dorcas* |
|  |  |  | *B. calyciflorus spinosa* |
|  | Lake Porcu |  | *B. calyciflorus dorcas* |
|  |  |  | *B. calyciflorus spinosa* |
| United States | Lake Hodges | Nogrady (1983) | *B. calyciflorus* *anuraeiformis* |
| Serbia | Donau Theis | Pujin et al. (1984) | *B. calyciflorus dorcas* |
|  |  |  | *B. calyciflorus* *amphiceros* |
|  |  |  | *B. calyciflorus* *calyciflorus* |
| India | Kailasaga | Sharma & Saksena (1984) | *B. calyciflorus* f. *dorcas* |
|  |  |  | *B. calyciflorus* f. *amphiceros* |
|  |  |  | *B. calyciflorus* f. *calyciflorus* |
| Republic of Senegal |  | De Rider (1985) | *B. calyciflorus* f. *amphiceros* |
| Mauritania | Mauritania | De Ridder (1987) | *B. calyciflorus* f. *amphiceros* |
|  |  |  | *B. calyciflorus* f. *anuraeiformis* |
| Australia |  | Koste & Shiel (1987) | *B. calyciflorus* f. *amphiceros* |
|  |  |  | *B.* *calyciflorus* *calyciflorus* |
|  |  |  | *B. calyciflorus* f. *dorcas* |
|  |  |  | *B. calyciflorus gigantea* |
|  |  |  | *B. calyciflorus spinosus* |
| Brazil | São Francisco River | Neumann-Leitão & Nogueira-Paranhos (1987) | *B. calyciflorus* f. *anuraeiformis* |
|  |  |  | *B. calyciflorus calyciflorus* |
| Spain | Guadalquivie River | Guisante & Toja (1988) | *B. calyciflorus* f. *anuraeiformis* |
| Australia | Solomon Dam | Hawkins (1988) | *B. calyciflorus* f. *anuraeiformis* |
| Soudan |  | De Ridder (1989) | *B. calyciflorus* f. *amphiceros* |
| Venezuela | Orinoco River | Vásquez & Rey (1989) | *B. calyciflorus* f. *amphiceros* |
|  |  |  | *B. calyciflorus calyciflorus* |
| Spain | [L' Albufera de València](http://www.visitvalencia.com/en/what-to-visit-valencia/albufera-natural-park) | Alfonso & Miracle (1990) | *B. calyciflorus* f. *anuraeiformis* |
|  |  |  | *B. calyciflorus* f. *amphiceros* |
|  |  |  | *B. calyciflorus calyciflorus* |
| France | Geneva Lake | Balvay & Laurent (1990) | *B. calyciflorus* var. *anuraeiformis* |
| Spain | Torrent de Llunach | De Manuel (1990) | *B. calyciflorus* f. *dorcas* |
| Brazil | Suape | Neumann-Leitão (1990) | *B. calyciflorus calyciflorus* |
|  |  |  | *B. calyciflorus* f. *anuraeiformis* |
| Greece | Volvi Lake | Zarfdjian et al. (1990) | *B. calyciflorus* f. *anuraeiformis* |
| Argentina | Reconquista River | Kuczynski (1991) | *B. calyciflorus* f. *amphiceros* |
|  |  |  | *B. calyciflorus* f. *anuraeiformis* |
| Singapore | MacRitchie Reservoir | Sudzuki (1991) | *B. calyciflorus dorcas* |
|  | Singapore Science Centre |  |  |
| Taiwan | Cheng Ching Hu |  |  |
|  | Jih Yueh Tan |  |  |
|  | Oriental region |  |  |
| Singapore | East coast Park pond |  | *B. calyciflorus borgerti* |
| Taiwan | Cheng Ching Hu pond |  |  |
| Republic of Mali | Mali | De Ridder (1992) | *B. calyciflorus* var. *amphiceros* |
| Negeria | Niger-Sokoto River | Jeje & Fernando (1992) | *B. calyciflorus calyciflorus* |
| Brazil | Suape | Neumann-Leitão et al. (1992) | *B. calyciflorus* *anuraeiformis* |
|  |  |  | *B. calyciflorus calyciflorus* |
| India | Raj Dlghi pond | Sharma & Dudani (1992) | *B. calyciflorus dorcas* |
|  | Harahi pond |  |  |
|  | Raj Dlghi pond |  | *B. calyciflorus* *anuraeiformis* |
|  | Harahi pond |  |  |
| India | Darbhanga City | Sharma et al. (1992) | *B. calyciflorus* *anuraeiformis* |
|  |  |  | *B. calyciflorus calyciflorus* |
| Japan | Ishigaki Jima | Sudzuki (1992) | *B. calyciflorus dorcas* |
|  | Yonaguni Jima |  |  |
|  | Okinawa mainland |  |  |
|  | Sesoko Jima |  |  |
|  |  |  | *B. calyciflorus* f. *amphiceros* |
|  | Taketomi Jima |  |  |
|  |  |  | *B. calyciflorus f. spinus* |
| Ukraine | Sasyk Reservoir | Koval’chuk & Parchuk (1993) | *B. calyciflorus calyciflorus* |
|  |  |  | *B. calyciflorus dorcas* |
|  |  |  | *B. calyciflorus* *anuraeiformis* |
|  |  |  | *B. calyciflorus* *amphiceros* |
|  |  |  | *B. calyciflorus spinosus* |
| Mexico | Lake Chapultepec | Rico-Martínez & Silva-Briano (1993) | *B. calyciflorus* f. *anuraeiformis* |
|  | Presidente Calles Dam |  | *B. calyciflorus* f. *amphiceros* |
| Nigeria | Festac Creek | Egborge (1994) | *B. calyciflorus* *amphiceros* |
| Republic of Botswana | Okavango Delta | Cronberg et al. (1995) | *B. calyciflorus* f. *amphiceros* |
|  |  |  | *B. calyciflorus* f. *anuraeiformis* |
| Hungary | Tisza river & tributaries | Gulyás et al. (1995) | *B. calyciflorus dorcas* |
|  |  |  | *B. calyciflorus spinosus* |
|  |  |  | *B. calyciflorus* *amphiceros* |
|  |  |  | *B. calyciflorus anuraeiformis* |
|  |  |  | *B. calyciflorus calyciflorus* |
| Estonia | Vortsjarv | Haberman (1995) | *B. calyciflorus anuraeiformis* |
|  |  |  | *B. calyciflorus calyciflorus* |
| Indonesia |  | Kutikova & Fernando (1995) | *B. calyciflorus* f. *amphiceros* |
| Philippines |  |  |  |
| Costa Rica |  |  |  |
| Venezuela |  |  |  |
| Bangladesh. |  |  | *B. calyciflorus borgerti* |
| Malaysia: |  |  |  |
| Ethiopia | Awasa Lake |  |  |
| Venezuela | Río Guasare-Limón | López & Ochoa (1995) | *B. calyciflorus* f. *amphiceros* |
| Thailand | Northeast Tailand | Sanoamuang et al. (1995) | *B. calyciflorus* f. *amphiceros* |
| Germany | Mühlweiher | Krause (1996) | *B. calyciflorus* *amphiceros* |
| Estonia | Lake Peipsi-Pihkva | Mäemets et al. (1996) | *B. calyciflorus anuraeiformis* |
|  |  |  | *B. calyciflorus calyciflorus* |
| Serbia | Tisza river | Pujin et al. (1996) | *B. calyciflorus* f. *amphiceros* |
|  |  |  | *B. calyciflorus* f. *anuraeiformis* |
| Russia | Lake Ladoga | Telesh (1996) | *B. calyciflorus spinosus* |
|  |  |  | *B. calyciflorus* *dorcas* |
|  |  |  | *B. calyciflorus calyciflorus* |
| Spain | Mar de Ontígola | Velasco et al. (1996) | *B. calyciflorus* f. *anuraeiformis* |
| Estonia-Russia | Lake Peipsi | Virro (1996) | *B. calyciflorus* *anuraeiformis* |
| Japan | Lake Ikeda | Baloch et al. (1998) | *B. calyciflorus* f. *dorcas* |
| Brazil | Rio Goiana | De Souza et al. (1998) | *B. calyciflorus calyciflorus* |
|  |  |  | *B. calyciflorus* f. *anuraeiformis* |
| Germany | Hahnöfer Nebenelbe | Holst et al. (1998) | *B. calyciflorus* f. *amphiceros* |
|  |  |  | *B. calyciflorus* f. *anuraeiformis* |
| Australia | Hawkesbury-Nepean River | Kobayashi et al. (1998) | *B. calyciflorus* *anuraeiformis* |
|  |  |  | *B. calyciflorus* *amphiceros* |
| China | Hainan | Koste & Zhuge (1998) | *B. calyciflorus calyciflorus* |
|  |  |  | *B. calyciflorus* *amphiceros* |
|  |  |  | *B. calyciflorus* *anuraeiformis* |
|  |  |  | *B. calyciflorus* *borgerti* |
|  |  |  | *B. calyciflorus* *dorcas* |
| Germany | Dümmers | Leutbecher & Koste (1998) | *B. calyciflorus* var. *dorcas* |
| Brazil | Ipojuca river | Neumann-Leitão & Matsumura-Tundisi (1998) | *B. calyciflorus anuraeiformis* |
|  |  |  | *B. calyciflorus* *calyciflorus* |
| Mongolia | Bayannor Meng | Rong et al. (1998) | *B. calyciflorus* f. *amphiceros* |
|  | Huhehot City |  |  |
|  | Hulunbeir Meng |  |  |
|  | Ulanchabu Meng |  |  |
|  | Xilinguoler Meng |  |  |
|  | Yingan Meng |  |  |
| Algeria | Guerbes-Senhadja | Samraoui et al. (1998) | *B. calyciflorus* f. *amphiceros* |
| Poland | Lake Gardno | Paturej & Jabłonska (1999) | *B. calyciflorus amphiceros* |
|  |  |  | *B. calyciflorus calyciflorus* |
| Spain | Spanish reservoirs | Barrabin (2000) | *B. calyciflorus* f. *dorcas* |
|  |  |  | *B. calyciflorus* f. *amphiceros* |
| Hungary | Körös-Maros National Park | Gulyás (2000) | *B. calyciflorus dorcas* |
|  |  |  | *B. calyciflorus anuraeiformis* |
|  |  |  | *B. calyciflorus spinosus* |
|  |  |  | *B. calyciflorus* *amphiceros* |
|  |  |  | *B. calyciflorus calyciflorus* |
| Germany | Teiche in der Vegetationsgeschichtlichen Abteilung | Krause (2000) | *B. calyciflorus* *amphiceros* |
| Bulgaria | Shabla lake | Kovachev & Hainadjieva (2000) | *B. calyciflorus dorcas* |
|  |  |  | *B. calyciflorus calyciflorus* |
|  | Ezerets lake |  | *B. calyciflorus dorcas* |
|  |  |  | *B. calyciflorus calyciflorus* |
|  | Durankulak lake |  | *B. calyciflorus dorcas* |
|  |  |  | *B. calyciflorus calyciflorus* |
|  | Orlovo Swamp |  | *B. calyciflorus calyciflorus* |
| Spain | Estany de Cullera lagoon | Oltra & Miracle (2000) | *B. calyciflorus* *anuraeiformis* |
|  |  |  | *B. calyciflorus* *amphiceros* |
| Spain | Tablas de Daimiel National Park | Ortega-Mayagoitia et al. (2000) | *B. calyciflorus* *anuraeiformis* |
| Ukraine | Dnieper River | Parchuk & Klochenko (2000) | *B. calyciflorus dorcas* |
|  |  |  | *B. calyciflorus dorcas spinosus* |
|  |  |  | *B. calyciflorus spinosus* |
|  |  |  | *B. calyciflorus* *calyciflorus* |
|  |  |  | *B. calyciflorus anuraeiformis* |
|  |  |  | *B. calyciflorus* *amphiceros* |
| India | Tripura State | Sharma & Sharma (2000) | *B. calyciflorus* f. *dorcas* |
|  |  |  | *B. calyciflorus* f. *anuraeiformis* |
| Italy | Orta Lake | Bonacina & Pasteris (2001) | *B. calyciflorus* f. *anuraeiformis* |
|  |  |  | *B. calyciflorus* f. *amphiceros* |
| Bulgaria | Vaja Lake | Pandourski (2001) | *B. calyciflorus calyciflorus* |
|  |  |  | *B. calyciflorus anuraeiformis* |
|  |  |  | *B. calyciflorus dorcas* |
| Turkey | Asi River | Bozkurt et al. (2002) | *B. calyciflorus* *anuraeiformis* |
|  |  |  | *B. calyciflorus* *amphiceros* |
| Nigeria | Ogunpa River | Akin-Oriola (2003) | *B. calyciflorus* *anuraeiformis* |
|  | Ona River |  |  |
| Brazil | Rio Mogi-Guaçu | Eler et al. (2003) | *B. calyciflorus* var. *dorcas* |
|  |  |  | *B. calyciflorus* f. *amphiceros* |
| Lithuania | Curonian Lagoon | Pliūraitè (2003) | *B. calyciflorus* *amphiceros* |
|  |  |  | *B. calyciflorus spinosus* |
| Turkey | Yarseli Dam Lake | Bozkurt et al. (2004) | *B. calyciflorus* *amphiceros* |
|  |  |  | *B. calyciflorus* *anuraeiformis* |
| Greece | Trichonis Lake | Kehayias et al. (2004) | *B. calyciflorus* f. *anuraeiformis* |
| Brazil | Lago Amapa | Keppeler & Hardy (2004) | *B. calyciflorus* *anuraeiformis* |
| Brazil | Jundiaí Reservoir | Lucinda et al. (2004) | *B. calyciflorus* f. *anuraeiformis* |
|  | Billings -Taquacetuba |  |  |
| Greece | Lake Koroneia | Michaloudi & Kostecka (2004) | *B. calyciflorus* f. *dorcas* |
|  |  |  | *B. calyciflorus* f. *anuraeiformis* |
|  |  |  | *B. calyciflorus* f. *amphiceros* |
| Bulgaria | Srebarna Lake | Pehlivanov et al. (2004) | *B. calyciflorus dorcas* |
|  |  |  | *B. calyciflorus* *anuraeiformis* |
|  |  |  | *B. calyciflorus* *amphiceros* |
|  |  |  | *B. calyciflorus calyciflorus* |
|  |  |  | *B. calyciflorus* *spinosus* |
| Pakistan | Rawal Lake | Baloch et al. (2005) | *B. calyciflorus* f. *dorcas* |
|  |  |  | *B. calyciflorus* f. *amphiceros* |
| Romania | Dunăreni Lake | Dinu et al. (2005) | *B. calyciflorus* f. *amphiceros* |
|  |  |  | *B. calyciflorus calyciflorus* |
| India | Kondakarla Lake | Chandrasekhar & Siddiqi (2005) | *B. calyciflorus* var. *dorcas* |
| Serbia | Međuvršje Reservoir | Ostojić & Simić (2005) | *B. calyciflorus* *anuraeiformis* |
|  |  |  | *B. calyciflorus calyciflorus* |
| Estonia | Lake Võrtsjärv | Virro & Haberman (2005) | *B. calyciflorus* f. *anuraeiformis* |
|  |  |  | *B. calyciflorus* f. *amphiceros* |
| Brazil | Tapacurá reservoir | Almeida et al. (2006) | *B. calyciflorus* *anuraeiformis* |
|  |  |  | *B. calyciflorus calyciflorus* |
| Brazil | Paraguay River | Frutos et al. (2006) | *B. calyciflorus* f. *dorcas* |
|  |  |  | *B. calyciflorus* f. *amphyceros* |
| Russia | Baikal Lake | Melnik et al. (2006) | *B. calyciflorus* *amphiceros* |
|  |  |  | *B. calyciflorus* *anuraeiformis* |
|  |  |  | *B. calyciflorus spinosus* |
|  |  |  | *B. calyciflorus calyciflorus* |
| Republic of Macedonia | Dojran Lake | Lokoska et al. (2006) | *B. calyciflorus* *amphiceros* |
| India | Tamilnadu | Raghunathan & Suresh Kumar (2006) | *B. calyciflorus* f. *dorcas* |
|  |  |  | *B. calyciflorus* f. *amphiceros* |
|  |  |  | *B. calyciflorus* f. *borgerti* |
|  |  |  | *B. calyciflorus* var. *anuraeiformis* |
| Hungary | Danube | Schöll (2006) | *B. calyciflorus* f. *dorcas* |
|  |  |  | *B. calyciflorus calyciflorus* |
|  |  |  | *B. calyciflorus* f. *anuraeiformis* |
|  |  |  | *B. calyciflorus* f. *amphiceros* |
|  |  |  | *B. calyciflorus* f. *spinosus* |
| Spain | Estanque del Retiro Madrid | Velasco (2006) | *B. calyciflorus* f. *amphiceros* |
|  | Canto del Pico 5 |  |  |
|  | Embalse Los Peñascales |  |  |
|  | Laguna de San Juan |  | *B. calyciflorus* f. *anuraeiformis* |
|  |  |  | *B. calyciflorus calyciflorus* |
|  | Laguna de Ontígola |  |  |
|  | Laguna de las Madres |  |  |
|  | Embalse Los Peñascales |  |  |
|  | Matachiviles |  |  |
| France | Rhône-Alpes | Balvay (2007) | *B. calyciflorus calyciflorus* |
|  |  |  | *B. calyciflorus* f. *amphiceros* |
|  |  |  | *B. calyciflorus* f. *anuraeiformis* |
|  |  |  | *B. calyciflorus* var*. dorcas* |
| Poland | Lake Gosawskie | Bogacka - Kapusta (2007) | *B. calyciflorus* var. *dorcas* |
|  |  |  | *B. calyciflorus* f. *anuraeiformis* |
|  |  |  | *B. calyciflorus* f. *amphiceros* |
|  |  |  |  |
| Falkland Islands | Swan Inlet pond | Dartnall & Hollwedel (2007) | *B. calyciflorus* *anuraeiformis* |
|  | Gull Island pond |  | *B. calyciflorus calyciflorus* |
| Bulgaria | Struma River – Pchelina Reservoir | Kozuharov et al. (2007) | *B. calyciflorus dorcas* |
|  |  |  | *B. calyciflorus* *amphiceros* |
| India | East Calcutta Wetlands | Mukhopadhyay et al. (2007) | *B. calyciflorus dorcas* f. *spinosa* |
| India | Tamil Nadu | Raghunathan & Valarmathi (2007) | *B. calyciflorus* f. *borgerti* |
| Hungary | Ráckeve-Soroksár Danube | Vadadi-Fülöp et al. (2007) | *B. calyciflorus* *amphiceros* |
|  |  |  | *B. calyciflorus* *anuraeiformis* |
|  |  |  | *B. calyciflorus calyciflorus* |
| Greece | Trichonis Lake | Doulka & Kehayias (2008) | *B. calyciflorus* f. *anuraeiformis* |
| India | Dwarkeswar | Pradhan & Chakraborty (2008) | *B. calyciflorus* f. *anuraeiformis* |
|  |  |  | *B. calyciflorus* f. *amphiceros* |
|  |  |  | *B. calyciflorus* f. *dorcus* |
|  |  |  | *B. calyciflorus* f. *borgerti* |
|  | Kansai |  | *B. calyciflorus* f. *anuraeiformis* |
| Federal Democratic Republic of Nepal | Siddha Pokhari | Vaidya & Yadav (2008) | *B. calyciflorus* var. *dorcas* |
|  |  |  | *B. calyciflorus* f. *annuraeiformis* |
|  |  |  | *B. calyciflorus* *amphiceros* |
|  | Nag Pokhari |  | *B. calyciflorus* var. *dorcas* |
|  |  |  | *B. calyciflorus* f. *annuraeiformis* |
|  | Godavari Fish Pond |  | *B. calyciflorus* *amphiceros* |
|  |  |  | *B. calyciflorus* f. *annuraeiformis* |
|  | Taudah Lake |  | *B. calyciflorus* *amphiceros* |
| Spain | Castellón | Sancho & Ramia (2008) | *B. calyciflorus* f. *anuraeiformis* |
| Argentina | San Miguel del Monte | Benítez & Claps (2009) | *B. calyciflorus* *amphiceros* |
|  |  |  | *B. calyciflorus calyciflorus* |
| Germany | Lower Rhine | Friedrich & Pohlmann (2009) | *B. calyciflorus* *amphiceros* |
| Brazil | Paraná River | Lansac-Tôha (2009) | *B. calyciflorus spinosus* |
| Nigeria | Ehoma Lake | Okogwu (2009) | *B. calyciflorus dorcas* |
|  |  |  | *B. calyciflorus* *calyciflorus* |
| Hungary | Duna-Dráva National Park | Schöll (2009) | *B. calyciflorus dorcas* |
|  |  |  | *B. calyciflorus* *anuraeiformis* |
|  |  |  | *B. calyciflorus calyciflorus* |
|  |  |  | *B. calyciflorus spinosus* |
|  |  |  | *B. calyciflorus* *amphiceros* |
| Hungary | Danube | Schöll & Kiss (2009) | *B. calyciflorus dorcas* |
|  |  |  | *B. calyciflorus* *anuraeiformis* |
|  |  |  | *B. calyciflorus* *amphiceros* |
|  |  |  | *B. calyciflorus calyciflorus* |
|  |  |  | *B. calyciflorus spinosus* |
| Ukraine | Pripyat’ River | Semenova (2009) | *B. calyciflorus* *anuraeiformis* |
|  |  |  | *B. calyciflorus* *amphiceros* |
|  |  |  | *B. calyciflorus calyciflorus* |
|  |  |  | *B. calyciflorus* *spinosus* |
|  | Kiev Reservoir |  | *B. calyciflorus* *anuraeiformis* |
|  |  |  | *B. calyciflorus* *spinosus* |
| Romania | Danube | Sundri & Gomoiu (2009) | *B. calyciflorus* var. *amphiceros* |
|  |  |  | *B. calyciflorus* var. *pala* |
| Italy | Ca’ Morta Lake | Tavernini et al. (2009) | *B. calyciflorus* f. *amphiceros* |
| Russia | Middle Daugava | Deksne et al. (2010) | *B. calyciflorus* *amphiceros* |
|  |  |  | *B. calyciflorus calyciflorus* |
|  |  |  | *B. calyciflorus* *anuraeiformis* |
|  |  |  | *B. pala* |
| India | Pocharam lake | Chandrasekhar (2010) | *B. calyciflorus* var. *dorcas* |
|  |  |  | *B. calyciflorus* f. *borgerti* |
|  |  |  | *B. calyciflorus* f. *anuraeiformis* |
| Hungary | Kis-Duna | Móra et al. (2010) | *B. calyciflorus* *amphiceros* |
|  |  |  | *B. calyciflorus* *anuraeiformis* |
|  |  |  | *B. calyciflorus calyciflorus* |
| Romania | South-West Dobrudja | Romanescu et al. (2010) | *B. calyciflorus* f. *amphiceros* |
|  |  |  | *B. calyciflorus* var. *pala* |
| Nigeria | Ehoma Lake | Okogwu et al. (2010) | *B. calyciflorus dorcas* |
|  |  |  | *B. calyciflorus calyciflorus* |
| Brazil | Mossoró Rio | Serpe et al. (2010) | *B. calyciflorus* *amphiceros* |
| China | Yuehu Lake | Zhang et al. (2010) | *B. calyciflorus* *amphiceros* |
| Argentina | Laguna Grande | Chaparro et al. (2011) | *B. calyciflorus calyciflorus* |
| Belarus & Latvia | Daugava River | Deksne (2011) | *B. pala* |
| Latvia | Daugava River | Deksne et al. (2011a) | *B. calyciflorus calyciflorus* |
|  |  | Deksne et al. (2011b) | *B. pala* |
| Belarus & Latvia | Daugava River | Deksne & Škute (2011) | *B. calyciflorus calyciflorus* |
| Algeria | Lake Boukourdane | Hamaidi et al. (2011) | *B. calyciflorus calyciflorus* |
| Ukraine | West forest-steppe of Ukraine | Ivanets (2011) | *B. calyciflorus* *amphiceros* |
|  |  |  | *B. calyciflorus calyciflorus* |
|  |  |  | *B. calyciflorus spinosus* |
| Brazil | Paranoá Lake | Padovesi-Fonseca et al. (2011) | *B. calyciflorus* *amphiceros* |
|  |  |  | *B. calyciflorus calyciflorus* |
| Brazil | São Paulo | Souza-Suares et al. (2011) | *B. calyciflorus calyciflorus* |
| France | Hérault | Balvay (2012) | *B. calyciflorus* var. *dorcas* |
|  |  |  | *B. calyciflorus* f. *anuraeiformis* |
| India | River Kapila | Farshad & Venkataramana (2012) | *B. calyciflorus dorcas* |
|  |  |  | *B. calyciflorus dorcas* f. *spinosa* |
| Republic of Macedonia | Dojran Lake | Tasevska et al. (2012) | *B. calyciflorus* f. *amphiceros* |
| Sri Lanka | Hambantota Port | Wijetunge & Ranatunga (2012) | *B. calyciflorus calyciflorus* |
| Nigeria | Ohana Lake | Ajah (2013) | *B. calyciflorus dorcas* |
| Thailand |  | Athibai et al. (2013) | *B. calyciflorus* f. *anuraeiformis* |
|  |  |  | *B. calyciflorus* f. *amphiceros* |
| Greece | Lysimachia Lake | Chalkia & Kehayias (2013) | *B. calyciflorus* f. *anuraeiformis* |
| India | Nature Park at Kolkata | Chitra (2013) | *B. calyciflorus* f. *dorcas* |
|  |  |  | *B. calyciflorus* f. *anuraeiformis* |
|  | Captain Bherry |  |  |
|  | Bhagajatin Lake |  |  |
|  | Nalban Lake |  |  |
|  | Hooghly RGBG |  |  |
| Algeria | Boukourdane lake | Hamaidi-Chergui et al. (2013) | *B. calyciflorus calyciflorus* |
| India | Andhra Pradesh | Karuthapandi et al. (2013) | *B. calyciflorus calyciflorus* |
| Hungary | Lake Balaton | Körmendi (2013) | *B. calyciflorus* f. *dorcas* |
|  |  |  | *B. calyciflorus* f. *anuraeiformis* |
|  |  |  | *B. calyciflorus* f. *amphiceros* |
|  |  |  | *B. calyciflorus* f. *spinosus* |
|  |  |  | *B. calyciflorus* f. *calyciflorus* |
| Romania | Sfântu Gheorghe, Danube | Parpală et al. (2013) | *B. calyciflorus dorcas* |
| India | Deepor Beel | Sharma & Sharma (2013) | *B. calyciflorus* f. *dorcas* |
|  |  |  | *B. calyciflorus* f. *anuraeiformis* |
|  |  |  | *B. calyciflorus* f. *amphiceros* |
|  |  |  | *B. calyciflorus borgerti* |
| Finland | Finland | Silfverberg (2013) | *B. calyciflorus dorcas* |
|  |  |  | *B. calyciflorus amphiceros* |
|  |  |  | *B. calyciflorus pala* |
| Romania | Danube | Sundri (2013) | *B. calyciflorus* var. *amphiceros* |
| Kazakhstan | Lebazh’e | Yermolaeva (2013) | *B. calyciflorus* *anuraeiformis* |
| Romania | Danube | Zinevici et al. (2013) | *B. calyciflorus dorcas* |
|  |  |  | *B. calyciflorus dorcas spinosa* |
|  |  |  | *B. calyciflorus* *anuraeiformis* |
|  |  |  | *B. calyciflorus* *amphiceros* |
|  |  |  | *B. calyciflorus calyciflorus* |
| Russia | Novosibirsk Reservoir | Dvurechenskaya & Yermolaeva (2014) | *B. calyciflorus calyciflorus* |
| Romania | Sfântu Gheorghe | Florescu & Moldoveanu (2014) | *B. calyciflorus dorcas* |
| Greece | Trichonis Lake | Kehayias et al. (2014) | *B. calyciflorus* f. *anuraeiformis* |
|  | Amvrakia Lake |  |  |
|  | Lysimachia Lake |  |  |
|  | Stratos Reservoir |  |  |
| Italy | San Lanfranco wetland | Paganelli et al. (2014) | *B. calyciflorus* *amphiceros* |
|  |  |  | *B. calyciflorus* *anuraeiformis* |
| India | Northeast India | Sharma & Sharma (2014a) | *B. calyciflorus* f. *dorcas* |
|  |  |  | *B. calyciflorus* f. *anuraeiformis* |
|  |  |  | *B. calyciflorus* f. *amphiceros* |
| India | India | Sharma & Sharma (2014b) | *B. calyciflorus* f. *dorcas* |
|  |  |  | *B. calyciflorus* f. *anuraeiformis* |
|  |  |  | *B. calyciflorus* f. *amphiceros* |
| Albania | Shkodra Lake | Shumka (2014) | *B. calyciflorus* f. *amphiceros* |
| India |  | Sinha (2014) | *B. calyciflorus calyciflorus* |
| Belarus, Lithuania | Lake Drūkšiai | Vezhnavets et al. (2014) | *B. calyciflorus* *amphiceros* |
|  |  |  | *B. calyciflorus* *anuraeiformis* |
|  |  |  | *B. calyciflorus calyciflorus* |
| Iraq | Tigris River | Abdulwahab & Rabee (2015) | *B. calyciflorus* *amphiceros* |
|  |  |  | *B. calyciflorus calyciflorus* |
| Brazil | Tucuruí reservoir | Bezerra et al. (2015) | *B. calyciflorus* *anuraeiformis* |
|  |  |  | *B. calyciflorus calyciflorus* |
| India | Ambattur Lake | Bee et al. (2015) | *B. calyciflorus* f. *dorcas* |
| Iraq | Iraqi Southern Marshes | Hammadi et al. (2015) | *B. calyciflorus* f. *dorcas* |
|  |  |  | *B. calyciflorus* f. *amphiceros* |
|  |  |  | *B. calyciflorus* f*. spinosus* |
| Australia | Macquarie Marshes | Kobayashi et al. (2015) | *B. calyciflorus* *amphiceros* |
| Bulgaria | Zhrebchevo Reservoir | Kozuharov & Stanachkova (2015) | *B. calyciflorus dorcas* |
|  |  |  | *B. calyciflorus* *amphiceros* |
|  |  |  | *B. calyciflorus* *anuraeiformis* |
| Russia | Southern Urals | Rogozin et al. (2015) | *B. calyciflorus* *amphiceros* |
|  |  |  | *B. calyciflorus calyciflorus* |
| Pakistan | Lake Manchar | Saddozai et al. (2015) | *B. calyciflorus* f. *dorcas* |
|  |  |  | *B. calyciflorus* f. *anuraeiformis* |
|  |  |  | *B. calyciflorus* f. *amphiceros* |
|  |  |  | *B. calyciflorus calyciflorus* |
| Kazakhstan | Ishim River | Yermolaeva (2015) | *B. calyciflorus* *anuraeiformis* |
|  |  |  | *B. calyciflorus calyciflorus* |
| India | Veli | Anitha & George (2016) | *B. calyciflorus* f. *dorcas* |
|  |  |  | *B. calyciflorus* f. *anuraeiformis* |
|  |  |  | *B. calyciflorus* f. *amphiceros* |
|  |  |  | *B. calyciflorus borgerti* |
|  | Aakulam |  | *B. calyciflorus* f. *anuraeiformis* |
|  |  |  | *B. calyciflorus* f. *amphiceros* |
|  |  |  | *B. calyciflorus borgerti* |
|  | Thiruvallam |  | *B. calyciflorus* f. *dorcas* |
|  | Poonthura |  | *B. calyciflorus* f. *anuraeiformis* |
|  |  |  | *B. calyciflorus* f. *amphiceros* |
| Iraq |  | Hammadi (2016) | *B. calyciflorus* *amphiceros* |
|  |  |  | *B. calyciflorus calyciflorus* |
|  | Kalikar Pond | Murkute & Chavan (2016) | *B. calyciflorus* f. *amphiceros* |
|  |  |  | *B. calyciflorus* var. *borgerti* |
|  |  |  | *B. calyciflorus* var. *dorcus* |
|  | Lendra Pond |  | *B. calyciflorus* f. *amphiceros* |
|  |  |  | *B. calyciflorus* var. *borgerti* |
|  |  |  | *B. calyciflorus* var. *dorcus* |
|  | Kurza Pond |  | *B. calyciflorus* f. *amphiceros* |
|  |  |  | *B. calyciflorus* var. *borgerti* |
|  |  |  | *B. calyciflorus* var. *dorcus* |
|  | Barai Pond |  | *B. calyciflorus* f. *amphiceros* |
|  | Khed Pond |  |  |
| Iraq | Kufa Rive | Nashaat et al. (2016) | *B. calyciflorus* *amphiceros* |
|  |  |  | *B. calyciflorus calyciflorus* |
| India | Morna reservoir | Solanke & Dabhade (2016) | *B. calyciflorus* f. *amphiceros* |
|  |  |  | *B. calyciflorus* f. *borgerti* |
| Romania | Danube Delta | Tudor et al. (2016) | *B. calyciflorus* *amphiceros* |
| Russia | Minzelinskoe | Yermolaeva et al. (2016) | *B. calyciflorus* *dorcas* |
|  |  |  | *B. calyciflorus calyciflorus* |

**REFERENCES**

Abdulwahab, S. & Rabee A.M. (2015) Ecological factors affecting the distribution of the zooplankton community in the Tigris River at Baghdad region, Iraq. *Egyptian Journal of Aquatic Research*, 41, 187–196.

Aguesse, P. (1957) Complement a l’inventaire de la faune inverte´bre´e des eaux camarguaises. *Terre et Vie*, 11, 241–252.

Ajah, P.O. (2013) The limnology of Ohana Lake, a potential manmade aquaculture system in Nigeria. *Open Journal of Applied Sciences*, 3, 232–246

Akin-Oriola, G.A. (2003) Zooplankton association and environmental factors in Ogunpa and Ona rivers, Nigeria. *Revista de Biologia Tropical*, 51, 391–398.

Alfonso, M.T. & Miracle, M.R. (1990) Distribución espacial de las comunidades zooplanctônicas de la Albufera de Valencia. *Scientia gerundensis,* 16, 11–25.

Allen, W.E. (1920) Some micro-plankton from Salton Sea *Science*, 51 (1324), 487.

Almeida, V.L. dos S., Larrazábal, M.E.L. de, Moura, A. do N. & Melo Júnior, M. de. (2006) Rotifera das zonas limnética e litorânea do reservatório de Tapacurá, Pernambuco, Brasil. *Iheringia. Série Zoologia*, 96 (4), 445–451.

Anitha, P.S. & George, R.M. (2016) Contributions to the rotifer fauna of Kerala (India) with two new records and remarks on some species. *International Journal of Fauna and Biological Studies*, 3 (3), 113–118.

Apstein, C. (1907) Das Plancton im Colombo-See auf Ceylon. *Zoologische Jahrbücher, Abteilung für Systematik, Geographie und Biologie der Tiere*, 25, 201–244.

Arévalo, C. (1918a) Algunos rotíferos planktónicos de la Albufera de Valencia. *Anales del Instituto General Técnico de Valencia*, 8, 1–47.

Arévalo, C. (1918b) Datos para el conocimiento del plankton de agua dulce en Barcelona. *Treballs de la Societat Catalana de Biologia*, 6.

Athibai, S., Segers, H. & Sanoamuang, L. (2013) Diversity and distribution of Brachionidae (Rotifera) in Thailand, with a key to the species. *Journal of Limnology*, 72 (2), 345–360.

Aurich, H. J. 1993. Das Zooplankton einiger Seen des Chiemgaus. Seine lokale Verbreitung und

Verteilung im Raum und Zeit. Internationale Revue der gesamten Hydrobiologie 29: 2955354

Aurich, H. J. 1993. Das Zooplankton einiger Seen des Chiemgaus. Seine lokale Verbreitung und

Verteilung im Raum und Zeit. Internationale Revue der gesamten Hydrobiologie 29: 2955354

Aurich, H. J. 1993. Das Zooplankton einiger Seen des Chiemgaus. Seine lokale Verbreitung und

Verteilung im Raum und Zeit. Internationale Revue der gesamten Hydrobiologie 29: 2955354

Aurich, H. J. 1993. Das Zooplankton einiger Seen des Chiemgaus. Seine lokale Verbreitung und

Verteilung im Raum und Zeit. Internationale Revue der gesamten Hydrobiologie 29: 2955354

Aurich, H.J. (1933) Das zooplankton einiger seen des Chiemgaus. Seine lokale Verbreitung und Verteilung in Ram und Zeit. Internationale *Reveue der Gesamten Hydrobiologie und Hydrographie*, 29, 295–354.

Baloch, W.A., Maeda, H. & Saisho, T. (1998) Seasonal abundance and vertical distribution of zooplankton in Lake Ikeda, southern Japan. *Microbes and Environments*, 13, 1–8.

Baloch, W.A., Jafri, S.I.H. & Soomro, A.N. (2005) Spring Zooplankton Composition of Rawal Lake, Islamabad. *Sindh University Research Journal (Science Series)*, 37 (2), 41–46.

Balvay, G. & Laurent, M. (1990) Evolution qualitative à long terme des rotiféres du Lac Léman. *Aquatic Sciences*, 52 (2), 156–161.

Balvay, G. (2007) Le zooplancton des étangs de la Dombes (Ain). *Bulletin mensuel de la Société linnéenne de Lyon*, 77 (1-2), 6–16.

Balvay, G. (2012) Compléments à l’inventaire des rotifères et des microcrustacés de l’Hérault. *Annales de la Société d’Horticulture et d’Histoire Naturelle de l’Hérault*, 52 (3), 108–122.

Barrabin, J.M. (2000) The rotifers of Spanish reservoirs: Ecological, systematical and zoogeographical remarks. *Limnetica* 19, 91–167.

Bartoš, E. (1948) On the Bohemian species of the genus Pedalia Barrois. *Hydrobiologia*, 1, 63–77.

Bee, K.S., Chitra, J. & Malini, E. (2015) Studies on plankton diversity and water quality of Ambattur Lake, Tamil Nadu. *International Journal of Pure and Applied Zoology*, 3 (1), 31–36.

Bērzins, B. (l954) Zur Rotatorien Fauna Siziliens. Hydrobiologia, 6, 309–320.

Benítez, H.H. & Claps, M.C. (2009) Distribución horizontal y vertical del zooplancton en un ciclo diario en el litoral de una laguna pampásica. *Biología Acuática*, 26, 19–31.

Bezerra, M.F.C., Sena, B.A., Martinelli-Filho, J.E., Nakayama, L. & Ohash, O.M. (2015) Composição e variabilidade da comunidade de rotifera em um reservatório tropical. *Boletim do Instituto de Pesca*, 41(3), 493–506

Bogacka-Kapusta, E. (2007) Changes in the abundance, species richness, and size structure of zooplankton under the influence of environmental conditions in the shallow littoral zone of a heated lake. *Archives of Polish Fisheries*, 15 (4), 335–351.

Bonacina, C. & Pasteris, A. (2001) Zooplankton of Lake Orta after liming: an eleven years study. *Journal of Limnology,* 60, 101–109.

Bozkurt, A., Göksu, M.Z.L., Sarıhan, E. & Taşdemir, M. (2002) Asi Nehri rotifer faunası (Hatay, Türkiye). *E.Ü.Su Ürünleri Dergisi*, 19 (1–2), 63–67.

Bozkurt, A., Dural, M. & Yılmaz, A.B. (2004). Yarseli Baraj Gölünün (Hatay-Türkiye) bazı fiziko-kimyasal özellikleri ve zooplankton (rotifer, kladoser ve kopepod) faunası. *Ulusal Su Günleri*, 6–8.

Brehm, V. (1909) Über die Mikrofauna chinesischer und südasiatischer Süsswasserbecken. *Archiv für Hydrobiologie*, 4, 207–224.

Brown, H.P. & McDaniel, H.C. (1952) Preliminary survey of sedentary invertebrates in an Oklahoma pond. *Proceedings of the Oklahoma Academy of Science*, 33, 115–121.

Bryce, D.L. (1931) Report on the Rotifera: Mr. Omer-Cooper's investigation of the Abyssinian fresh wa-ters (Dr. Hugh Scott Expedition). *Proceedings of the Zoological Society of London*, 5, 865–878.

Buchner, H. (1937) Experimentelle Untersuchungen iiber den Generationswechsel der Riidertiere. *Zeitschrift für Induktive Abstammungs und Vererbungslehre*, 72, 1–49.

Buchner, H. & Mulzer, F. (1961) Untersuchungen über die variabilität der rädertiere. II. Der ablauf der variation im freien. *Zeitschrift für Morphologie und Ökologie der Tiere*, 50 (3), 330–374.

Chaparro, G., Marinone, M.C., Lombardo, R.J., Schiaffino, M.R., de Souza Guimarães, A. & O’Farrell, I. (2011) Zooplankton succession during extraordinary drought-floodncycles: a case study in a South America floodplain lake. *Limnologica*, 41, 371–381.

Chalkia, E. & Kehayias, G. (2013) Zooplankton and environmental factors of a recovering eutrophic lake (Lysimachia Lake, Western Greece). *Biologia* 68, 459–469.

Chandrasekhar, S.V.A. & Siddiqi, S.Z. (2005) Kondakarla lake, Andhra Pradesh - a taxo-ecological profile. *Records of the Zoological Survey of India*, 104 (3–4), 63–76.

Chandrasekhar, S.V.A. (2010) Zooplankton: Pocharam Lake. *In:* Editor-Director (ed). *Limnological and Faunistic Studies of Pocharam Lake,Nizamabad-Medak District, Andhra Pradesh, Wetland Ecosystem Series*, *13.* Director, Zoological Survey of India, Kolkata, pp. 29–35.

Chitra, J. (2013) Diversity, abundance and seasonal fluctuation of zooplankton from few wetlands in and around Kolkata. *Records Of The Zoological Survey Of India Occasional Paper No.*, 352, 1–47.

Cohn, F. (1862) Bemerkungen über Räderthiere. III. *Zeitschrift für wissenschaftliche Zoologie*, 12, 197–217.

Colditz, F.W., (1914) Beiträge zur Biologie des Mansfelder Sees mit besonderen Studien über das Zentrifugenplankton und seine Beziehungen zum Netzplankton der pelagischen Zone. *Zeitschrift für wissenschaftliche Zoologie,* 108, 521–630.

Collin, A., Dieffenbach, H., Sachse, R. & Voigt, M. (1912) Rotatoria und Gastrotricha. *Die Süßwasserfauna Deutschlands*, 14, 1–273.

Cronberg, G., Gieske, A., Martins, E., Prince Nengu, J. & Stenström, I.M. (1995) Hydrobiological Studies of the Okavango Delta and Kwando/Linyanti/Chobe River, Botswana I Surface Water Quality Analysis. *Botswana Notes and Records*, 27, 151–226.

Daday, E. (1883) Neue Beiträge zur Kenntnis der Rädertiere. *Mathematische und Naturwissenschaftliche Berichte aus Ungarn,* 1, 261–264.

Daday, E. (1901) Mikroskopische Süsswasserthiere (Zoologische Ergebnisse der dritten asiatischen Forschungsreise des Grafen Eug. Zichy). *Dritte asiatische Forschungsreise des Grafen Eugen Zichy*, 2, 375–479.

Damas, H. (1941) La faune de la Meuse belge. *Association Française pour l'Avancement des Sciences*, 150–159.

Dartnall, H.J.G. & Hollwedel, W. (2007) A limnological reconnaissance of the Falkland Islands; with particular reference to the waterfleas (Arthropoda: Anomopoda). *Journal of Natural History*, 41, 1259–1300.

De Beauchamp, P. (1932a) XIX.-Reports on the Percy Sladen expedition to some Rift Valley Lakes in Kenya in 1929.-III. Rotifères des Lacs de la Vallée du Rift. *Annals and Magazine of Natural. History*, 10, 158–165.

De Beauchamp, P. (1932b) Scientific rc?yults of the Cambridge Erpedition to the East African Lakes, 1930 -1. 6. Rotifbres et Gastrotriohcs. *Zoological Journal of the Linnean Society*, 38, 231–248.

De Beauchamp, P. (1939). Percy Sladen trust expedition to Lake Titicaca in 1937. V. Rotiferes et Turbellaries. *Transactions of the Linnean Society of London.* 1, 51–79.

De Manuel, J. (1990) Contribution to the knowledge of the rotifer fauna (Rotifera: Monogononta) from Minorcan inland waters (Balearic isles: Spain). *Limnetica*, 6, 119–130.

De Ridder, M. (1985) Contributions to the knowledge of African Rotifers. I . Rotifers from Senegal. *Hydrobiologia*, 120, 47–51.

De Ridder, M. (1987) Contribution to the knowledge of African rotifers : Rotifers from Mauritania (W.-Africa). *Hydrobiologia*, 150, 123–131.

De Ridder, M. (1989) Rotifers from Western Sudan. *Hydrobiologia*, 179, 205–209.

De Ridder, M. (1992) Contribution to the study of African rotifers: Rotifers from Mali. *Hydrobiologia*, 237 (2), 93–101.

De Souza, F.B.V.A., Neumann-Leitão, S. & Paranaguá, M.N. (1998) Rotifera do sistema estuarino do Rio Goiana, Pernambuco, Brasil. *Trabalhos em Oceanografia da Universidade Federal de Pernambuco*, 26, 31–62.

Decksbach, N. (1926) Studien über das Zooplankton des Petsjora-Beckens und der südlichen Nebenflüsse der Dwina. *Internationale Revue der gesamten Hydrobiologie und Hydrographie,* 14, 323–338.

Deksne, R., Škute, A. & Paidere, J. (2010) Changes in structure of zooplankton communities in the Middle Daugava (Western Dvina) over the last five decades. *Acta Zoologica Lituanica*, 20 (3), 190–208.

Deksne, R. (2011) Influence of wastewater on zooplankton communities in the Daugava River upstream and downstream of Daugavpils over the last 50 years. *Knowledge and Management of Aquatic Ecosystems*, 402, 07

Deksne, R. & Škute, A. (2011) The influence of ecohydrological factors on the cenosis of the Daugava River zooplankton. *Acta Zoologica Lituanica*, 21, 133–144.

Deksne, R., Škute, A. & Meinerte, A. (2011a) Seasonal changes in zooplankton community of the Daugava river. *Acta Biologica Universitatis Daugavpiliensis*, 11 (1), 61–75.

Deksne, R., Škute, A., Gruberts, D. & Paidere, J. (2011b) Effects of climate change on zooplankton community structure of the middle stretch of the Daugava river over the last 50 years. *Ecohydrology & Hydrobiology*, 11, 79–96.

Dieffenbach, H. & Sachse, R. (1912) Biologische Untersuchungen an Rädertieren in Teichgewässern. Internationale *Revue der gesamten Hydrobiologie und Hydrographie*, 4, 1–93.

Dinu, C., Radu, A. & Török, L. (2005) Data on the hydrobiological characteristics of Dunareni Lake (Constanta County). *Scientific Studies and Research, Biology, University of Bacau*, 10, 33–36.

Dolley, J.S. (1933) Preliminary notes on the biology of the St. Joseph River. *The American Midland Naturalist*, 14, 193–227.

Dönner, J. (1949) Höraella brehmi nov. gen. nov. sp. eine neue Rädertiere aus Indian. *Hydrobiologia*, 2, 304–328.

Doulka, E. & Kehayias, G. (2008) Spatial and temporal distribution of zooplankton in Lake Trichonis (Greece). *Journal of Natural History*, 42, 575–595.

Dunn, I.G. (1970) Recovery of a tropical pond zooplankton community after destruction by algal bloom. *Limnology and Oceanography*, 15, 373–379.

Dvurechenskaya, S.Y. & Yermolaeva, N.I. (2014) Interrelations between chemical composition of water and characteristics of zooplankton in the Novosibirsk reservoir. *Contemporary Problems of Ecology*, 7 (4), 465–473.

Ehrenberg, C.G. (1830) *Organisation, Systematik und geographisches Verhältnis der Infusionsthierchen. Zwei Vorträge in der Akademie der Wissenschaften zu Berlin gehalten in den Jahren 1828 [Die geographische Verbreitung der Infusionsthierchen in Nord-Afrika und West-Asien, beobachtet auf Hemprich und Ehrenbergs Reisen] und 1830 [Beiträge zur Kenntnis der Organisation der Infusorien und ihrer geographischen Verbreitung, besonders in Sibirien].* Druckerei der königlichen Akademie der Wissenschaften, Berlin, 108 pp.

Egborge, A.B.M. (1994) Salinity and the distribution of rotifers in the Lagos Harbour - Badagry Creek system, Nigeria. *Hydrobiologia*, 272, 95–104.

Ehrenberg, C.G. (1838) *Die Infusionsthierchen als vollkommene Organismen. Ein Blick in das tiefere organische Leben der Natur*. Verlag von Leopold Voss, Leipzig.

Eler, M.N., Pareschi, D.C., Espíndola, E.L.G. & Barbosa, D.S. (2003) Ocorrência de Rotifera e sua relação com o estado trófico da água em pesque-pague na bacia do rio Mogi-Guaçu – SP. *Boletim Técnico do CEPTA, Pirassununga*, 16, 41–56.

Farshad, H. & Venkataramana, G.V. (2012) Impact of physicochemical parameters of water on zooplankton diversity in Nanjangud Industrial Area, India. *International* *Research Journal of Environment Sciences*,1 (4), 37–42.

Fernando, C.H. & Zankai, N.P. (1981) The Rotifera of Malaysia and Singapore with remarks on some species. *Hydrobiologia*, 78, 205–219.

Florescu, L. & Moldoveanu, M. (2014) The rotifer communities seasonality from Sfântu Gheorghe branch (Danube Delta). *Ecoterra*, 11 (3), 20–27.

France, R.H. (1894) Beiträge zur Kenntnis der Rotatorienfauna Budapest's. *Természetrajzi Füzetek kiadja a Magyar nemzeti Múzeum*, 17: 166–184.

Friedrich, G. & Pohlmann, M. (2009) Long-term plankton studies at the lower Rhine/Germany. *Limnologica*, 39, 14–39.

Frutos, S.M., Poi de Neiff, A.S.G. & Neiff, J.J. (2006) Zooplankton of the Paraguay River: a comparison between sections and hydrological phases. *Annales de Limnologie - International Journal of Limnology*, 42 (2), 277–288.

Gilbert, J.J. & Waage, J.K. (1967) *Asplanchna*, *Asplanchna*-substance, and posterolateral spine length variation of the Rotifer *Brachionus Calyciflorus* in a natural environment. *Ecology*, 48 (6), 1027–1031.

Godeanu, S. & Zinevici, V. (1983) Composition, dynamics and production of Rotatoria in the plankton of some lakes of the Danube Delta. *Hydrobiologia*, 104, 247–257.

Gosse, P.H.A.L.S. (1851) XVIII.-A catalogue of Rotifera found in Britain; with descriptions of five new genera and thirty-two new species. *Annals and Magazine of Natural History*, 8 (45), 197–203.

Gray, E.A. (1953) The ecology of rotifers in Cambridgeshire. *Journal of Animal Ecology*, 22, 208–216.

Guisande, C. & Toja, J. (1988) The dynamics of various species of the genus Brachionus (Rotatoria) in the Guadalquivir River. *Archiv für Hydrobiologie*, 112, 579–595.

Gulyás, P. (2000) Cercetări asupra grupurilor Rotatoria şi Crustacea din apele de pe teritoriul Parcului Naţional Criş-Mureş. *Crisicum*, 3, 109–137.

Gulyás, P., Bancsi, I. & Zsuga, K.V. (1995) Rotatoria and Crustacea fauna of the Hungarian watercourses. *Miscellanea Zoologica Hungarica*, 10, 21–47.

Haberman, J. (1995) Dominant rotifers of Vortsjarv (Estonia). *Hydrobiologia*, 313/314, 313–317.

Hamaidi, F., Hamaidi, M.S., Benouaklil, F. & Saidi, F. (2011) The Rotifera fauna from Algeria. *Bioresearch Bulletin*, 1 (4).

Hamaidi-Chergui, F., Hamaidi, M.S., Errahman, M.B. & Benouaklil, F. (2013) Studies on biodiversity of rotifera in five artificial lakes in Algeria: Systematical and zoogeographical remarks. *Kragujevac Journal of Science*, 35, 115–138.

Hammadi, N.S., Salman, S.D. & Abbas, M.F. (2015) Rotifera of the Southern Iraqi Marshes, with a reference to the major zooplankton groups in the region. *Marsh Bulletin*, 10, 46–55.

Hammadi, N.S. (2016) An ecological survey of littoral Rotifera from some selected areas of Iraq: I. middle of Iraq. *Journal of Basrah Researches*, 42 (2), 46–55.

Harrison, A.D. (1962) Hydrobiological studies on alkaline and acid still waters in the Western Cape Province. *Transactions of the Royal Society of South Africa*, 36 (4), 213–244.

Hartmann, O. (1915) Rotatorien, Copepoden und Cladoceren aus der Bukowina. *Verhandlungen der Zoologisch-Botanischen Gesellschaft in Wien*, 65, 231–237.

Hauer, J. (1965) Zur Rotatorienfauna des Amazonasgebietes*. International Review of Hydrobiology*, 50 (3), 341–389.

Havinga, B. (1919) Studien over flora en fauna van het Zuidlaarder meer: bijdrage tot de kennis van de biologie der Nederlandsche meren. Groningen.

Hawkins, P.R. (1988) The zooplankton of a small tropical reservoir (Solomon Dam, north Queensland). *Hydrobiologia*, 157, 105–118.

Henry, G. (2013) On the vertical distribution of the plankton in Winona Lake. *Proceedings of the Indiana Academy of Science*, 77–92

Holst, H., Zimmermann, H., Kausch, H. & Koste, W. (1998) Temporal and special distribution of planktonic rotifers in the Elbe Estuary during spring. *Estuarine, Coastal and Shelf Science*, 47, 261–273.

Hudson, C.T. & Gosse, P.H. (1886) *The Rotifera; or wheel-animalcules, both British and foreign*. Longmans, Green, and Co., London.

Huss, H. (1913) Können die Cyclopiden intramolekular atmen? *Internationale Revue der gesamten Hydrobiologie und Hydrographie*, 6, 38.

Ivanets, O. (2011) Zooplankton of the water vegetation in the ponds of west forest-steppe of Ukraine. *Visnyk of the Lviv University.* *Series BIology*, 56, 148–156.

Jeje, C.Y. & Fernando, C.H. (1992) Zooplankton Associations in the Middle Niger-Sokoto Basin (Nigeria: West Africa). *Internationale Revue der gesamten Hydrobiologie und Hydrographie*, 77 (2), 237–253.

Karuthapandi, M., Rao, D.V. & Innocent, X. (2013) Freshwater rotifers of Andhra Pradesh – Checklist. *International Journal for Life Sciences and Educational Research*, 1(1), 1–13.

Kehayias, G., Michaloudi, E. & Bexi, A. (2004) Aspects on the seasonal dynamics and the vertical distribution of the crustacean zooplankton community and the Dreissena polymorpha larvae in Lake Trichonis. *Mediterranean Marine Science*, 5 (1), 5–17.

Kehayias, G., Chalkia, E. & Doulka, E. (2014) Zooplankton variation in five greek lakes. *In:* Kehayias, G. (ed). *Zooplankton.* Nova Science Publishers, New York, pp. 85–119.

Keppeler, E.C. & Hardy, E.R. (2004) Abundance and composition of Rotifera in an abandoned meander lake (Lago Amapá) in Rio Branco, Acre, Brazil. *Revista Brasileira de Zoologia*, 21 (2), 233-241.

Kertész, G. (1967) Längsprofiluntersuchungen des rotatorienplanktons im ungarischen abschnitt der Donau. *Opuscula Zoologica Budapest*, 7, 189–199.

Kobayashi, T., Shiel, R.J., Gibbs, P. & Dixon, P.I. (1998) Freshwater zooplankton in the Hawkesbury-Nepean River: comparison of community structure with other rivers. *Hydrobiologia*, 377, 133–145.

Kobayashi, T., Ralph, T.J., Ryder, D.S., Hunte, S.J., Shiel, R.J. & Segers, H. (2015) Spatial dissimilarities in plankton structure and function during flood pulses in a semi-arid floodplain wetland system. *Hydrobiologia*, 747, 19–31.

Körmendi, S. (2013) Halastavak hidrobiológiai vizsgálata a Balaton déli vízgyűjtőjén. *Acta Scientiarum Socialium*, 39, 95–111.

Koste, W. (1976) Über die Rädertierbestände (Rotatoria) der oberen und mittleren Hase in den Jahren 1966-1969. *Osnabrücker naturwissenschaftliche Mitteilungen*, 4, 191–263.

Koste, W. (1978) *Rotatoria. Die Rädertiere Mitteleuropas. Ein Bestimmungswerk, begründet von Max Voigt*. Überordnung Monogononta. Gebrüder Borntraeger, Berlin, Stuttgart.

Koste, W. & Shiel, R.J. (1980) Preliminary remarks on the characteristics of the rotifer fauna of Australia (Notogaea). *Hydrobiologia*, 73, 221–227.

Koste, W. & Shiel, R.J. (1987) Rotifera from Australian inland waters II. Epiphanidae and Brachionidae. *Invertebrate Taxonomy*, 7, 949–1021.

Koste, W. & Zhuge, Y. (1998) Zur Kenntnis der Rotatorienfauna (Rotifera) der Insel Hainan, China. Teil II. *Osnabrücker naturwissenschaftliche Mitteilungen*, 24, 183–222.

Kovachev, S. & Hainadjieva, V. (2000) The pelagic Zooplankton of several coastal lakes in North-East Bulgaria. *Lauterbornia*, 38, 63–66.

Koval'chuk, A. & Parchuk, G. (1993) Rotifers of Sasyk Reservoir and their role in the production and decomposition of organic matter. *Hydrobiological Journal*, 29 (1), 51–63.

Kozuharov, D., Evtimova, V. & Zaharieva, D. (2007) Long-term changes of zooplankton and dynamics of eutrophication in the polluted system of the Struma River - Pchelina Reservoir (South-West Bulgaria). *Acta Zoologica Bulgarica*, 59 (2), 191–202.

Kozuharov, D. & Stanachkova, M. (2015) Long-term changes in zooplankton community in Zhrebchevo Reservoir, Central Bulgaria. *Acta Zoologica Bulgarica*, 67 (4), 541–552.

Krampner, A. (1928) Wandlungen der Kruster- und Rotatorien-Fauna. [Inte*rnationale Revue der gesamten Hydrobiologie und Hydrographie*](https://onlinelibrary.wiley.com/journal/15222632a), 19, 281-288

Krause, L. (1996) Planktonbeobachtungen in heiligkreuztal. *Mitteilungen der Mikro AG Stuttgart e. V.*, 2–4, 1–7.

Krause, L. (2000) Kleingewässer in Hohenheim, Teil 1. Die Teiche in der Vegetationsgeschichtlichen Abteilung. *Mitteilungen der Mikro AG Stuttgart*, 1/2, 1–47.

Kuczynski, D. (1991) Rotifers from Reconquista River, Argentina: the genus Brachionus, with descriptions of new species. *Hydrobiologia,* 215, 135–152.

Künne, C. (1926) Zur rädertier-fauna des Seeburger sees. Zeitschrift für Morphologie und Ökologie der Tiere, 6 (2), 207–286.

Kutikova, L.A. & Fernando, C.H. (1995) Brachionus calyciflorus Pallas (Rotatoria) in inland waters of tropical latitudes. *Internationale Revue der gesamten Hydrobiologie und Hydrographie*, 80, 429–441.

Lansac-Tôha, F.A., Bonecker, C.C., Velho, L.F.M., Simões, N.R., Dias, J.D., Alves, G.M. & Takahashi, E.M. (2009) Biodiversity of zooplankton communities in the Upper Paraná River floodplain: interannual variation from long-term studies. *Brazilian Journal of Biology*, 69 (2), 539–549.

Leentvaar, P. (1967) *Een overzicht van de hydrobiologische toestand in de Vechtplassen in 1957 en 1958*. Rivon, Zeist.

Lefèvre, M. (1941) Recherches hydrobiologiques sur les rivières, mares et étangs du Domaine National de Rambouillet. Bulletin Français de Pisciculture, 122, 89–143.

Lehmann, C. & Westf, M. (1933) Beiträge zur Kenntnis der Fauna westdeutscher Gewässer. *Internationale Revue der gesamten Hydrobiologie und Hydrographie*, 29, 113–122.

Leutbecher, C. & Koste, W. (1998) Die Rotatorienfauna des Dümmers unter besonderer Berücksichtigung der sessilen Arten. Teil I. *Osnabrücker naturwissenschaftliche Mitteilungen*, 24, 223–255.

Löffler, H. (1961) Beitrage zur Kenntnis der Iranischen Binnegewasser. II. Regional- Limnologische Studie Mit besondere Berücksichtigung der Crustaceeenfauna. *Internationale Revue der gesamten Hydrobiologie und Hydrographie*, 46, 309–406.

Löffler, H. (1962) Ergebnisse der Zoologischen Nubien-Expedition 1962 Teil XVIII, Zur Binnenwasserfaunaeiniger Kleingewässer und Brunnen im nördlichen Sudan. *Annalen des Naturhistorischen Museums in Wien*, 66, 489–494.

Lokoska, L., Jordanoski, M., Veljanoska-Sarafiloska, E. & Taveska, O. (2006) *Water quality of Lake Dojran from biological and physical-chemical aspects. Conference on water observation and information system for decision support*, Ohrid, Macedonia 23-26 May 2006.

Lopez, C. & Ochoa, E. (1995) Rotiferos (Monogononta) de la Cuenca del Rio Guasare-Limon, Venezuela. *Revista de Biologia Tropical*, 43 (1-3), 189–193.

Lucinda, I., Moreno, I.H., Melão, M.G.G. & Matsumura-Tundisi, T. (2004) Rotifers in freshwater habitats in the upper Tietê river basin, São Paulo State, Brazil. *Acta Limnologica Brasiliensia*, 16 (3), 203–224.

Mäemets, A., Timm, M. & Nõges, T. (1996) Zooplankton of Lake Peipsi-Pihkva in 1909-1987. *Hydrobiologia*, 338, 105–112.

Mann, A.K. (1940) Über pelagische copepoden Turkischer Seen (mit Beriicksichtigung des iibrigen Blanktons). *International Revue der gesamten Hydrobiologie und Hydrographie*, 40, 1–87.

Marcuzzi, G. (1979) *European Ecosystems*. Junk, Hague-Boston-London.

Meissner, V. (1902) Zhivotnyj plankton reki Volgi pod Saratovom. *Trudy Saratovskogo obshchestva jestestvoispytatelej i liubitelej jestestvoznanija, Saratov*, 3, 1–69.

Melnik, N.G., Bondarenko, N.A., Belykh, O.I., Blinov, V.V., Ivanov, V.G., Korovyakova, I.V., Kostornova, T.Y., Lazarev, M.I., Logacheva, N.F., Pomazkova, G.I., Sherstyankin, P.P., Sorokovikova, L.M., Tolstikova, L.I. & Tereza, E.P. (2006) Distribution of pelagic invertebrates near thermal bar in Lake Baikal. *Hydrobiologia*, 568, 69–76.

Michaloudi, E. & Kostecka, M. (2004) Zooplankton of Lake Koroneia (Macedonia, Greece). *Biologia*, 59, 165–172.

Móra, A., Kálmán, Z., Soós, N., Tóth, A., Deák, C.S., Ambrus, A. & Csabai, Z. (2010) [Data to the aquatic invertebrate fauna of Kis-Duna (Kismaros) with first Hungarian records of three chironomid species](https://scholar.google.hu/citations?view_op=view_citation&hl=hu&user=7XO8tVMAAAAJ&citation_for_view=7XO8tVMAAAAJ:M0leSnx2MbUC). *Acta Biologica Debrecina Supplementum Oecologica Hungarica*, 21, 127–138.

Mukhopadhyay, S.K., Chattopadhyay, B., Goswami, A.R. & Chatterjee, A. (2007) [Spatial variations in zooplankton diversity in waters contaminated with composite effluents](https://scholar.google.gr/citations?view_op=view_citation&hl=en&user=0tL4OHsAAAAJ&citation_for_view=0tL4OHsAAAAJ:qjMakFHDy7sC). *Journal of Limnology*, 66 (2), 97–106.

Murray, J., 1906. The Rotifera of Scottish Lochs. *Transactions of the Royal Society of Edinburgh*. 45, 151–193.

Murkute, V.B. & Chavan, A.W. (2016) Report on rotifer diversity with reference to their role in eutrophication from lentic ecosystems at Bramhapuri, Dist: Chandrapur (M.S.) *India. International Journal of Researches in Biosciences, Agriculture and Technology*, 8–12.

Myers, F.J. (1942) The Rotatorian Fauna of the Pocono Plateau and Environs. *Proceedings of the Academy of Natural Sciences of Philadelphia*, 94, 251–285.

Nashaat, M.R., Rasheed, K.A. & Hassan, H.A. (2016) Rotifera Abundance and Species Diversity in Al-Kufa River, Iraq. *Global Journal of Science Frontier Research: (H) Environment & Earth Science*, 16 (5), 49–58.

Neumann-Leitão, S. & Nogueira-Paranhos, J.D. (1987) Zooplâncton do rio São Francisco, região nordeste do Brasil. *Trab Oceanography, Federal University of Pernambuco*, 20, 173–196.

Neumann-Leitão, S. (1990) Estudos taxonômicos dos Rotatoria da área estuarina-lagunar de Suape, Pernambuco (Brasil). *Tropical oceanography*, 21 (1).

Neumann-Leitão, S., Paranagua, M.N. & Valentin, J.L. (1992) The planktonic rotifers of the estuarine lagunar complex of Suape ( Pernambuco , Brazil ). *Hydrobiologia*, 232, 133–143.

Neumann-Leitão, S. & Matsumura-Tundisi, T. (1998) Dynamics of a perturbed Estuarine Zooplanktonic Community: Port of Suape, PE, Brazil. *Verhandlungen der Internationale Vereinigung für Limnologie, Stuttgart*, 26, 1981–1988.

Nipkow, F. (1958) Beobachtungen bei der Entwicklung des Dauereies von Brachionus calyciflorus Pallas. *Schweizerische Zeitschrift Fur Hydrologie*, 20, 186–194.

Nogrady, T. (1983) Some new and rare warmwater rotifers. *Hydrobiologia*, 106, 107–114.

Okogwu, I.O. (2009) Seasonal variations of species composition and abundance of zooplankton in Ehoma Lake, a floodplain lake in Nigeria. [*Revista de Biología Tropical*,](http://www.scielo.sa.cr/scielo.php?pid=0034-7744&script=sci_serial) 58 (1), 171–182.

Okogwu, I.O., Christopher, D.N. & Florence, A.O. (2010) Seasonal variation and diversity of rotifers in Ehomalake, Nigeria. *Journal of Environmental Biology*, 31, 533–537.

Oltra, R. & Miracle, M.R. (2000) Variación espacio-temporal de las poblaciones de rotíferos de la laguna meromíctica Estany de Cullera (Valencia). *Limnetica*, 19, 39–52.

Ortega-Mayagoitia, E. Armengol, X. & Rojo, C. (2000) Structure and dinamics of zooplankton in a semi-arid wetland, the national park Las Tablas de Daimiel (Spain). *Wetlans*, 20 (4), 629-638.

Otto, J.P. (1954) Over de flora en fauna van een oud baggerstortterrein en zijn omgeving (slot). *De Levende Natuur*, 57 (11), 214–219.

Ostojić, A. & Simić, V. (2005) Contribution to knowledge of zooplankton in the rivers of Serbia: Summer aspect of Rotatoria in Morava and Western Morava. *Kragujevac Journal of Science*, 27, 157–162.

Padovesi-Fonseca, C., de Mendonça-Galvão, L. & Andreoni-Batista, C. (2011) Rotifera, Paranoá reservoir, Brasília, central Brazil. *Check list the journal of biodiversity data*, 7 (3), 248–252.

Paganelli, D., Saltarelli, M. & Sconfietti, R. (2014) Seasonal evolution of the zooplankton community in two riverine wetlands of the Ticino River (Lombardy, Northern Italy). *Annales de Limnologie - International Journal of Limnology*, 50, 241–247.

Pandourski, I. (2001) Recherches hydrobiologiques des zones humides de la côte bulgare de la mer noire. I. Le lac de Vaja. *Rivista di Idrobiologia*, 40, 2–3.

Parchuk, G.V. & Klochenko, P.D. (2000) Comparative Characteristics of Zooplankton of the Dnieper Tributaries. *Hydrobiological Journal*, 36 (4), 15–33.

Parpală, L., Moldoveanu, M., Dumitrache, A. & Florescu, L. (2013) Dynamics of phyto- and zooplankton communities diversity of the Sfântu Gheorghe Arm under the anthropogenic impact. [*Romanian Journal of Biology - Zoology*](http://journals.indexcopernicus.com/Romanian+Journal+of+Biology+-+Zoology,p268,3.html), 58 (2), 123–139.

Paturej, E. & Jabłońska, I. (1999) The influence of water mass dynamics on the changes of invertebates number in Lake Gardno. *Baltic Costal Zone*, 3, 89–101

Peelen, R. (1974) Changes in the plankton of the estuarine area of the Haringvliet-Hollands Diep_Biesbosch in the S.W. Netherlands caused by the dams through Volkerak and Haringvliet. *Hydrobiological Bulletin*, 8, 190–200.

Pehlivanov, L., Tsavkova, V. & Naidenov, W. (2004) The metazoan plankton of the Biosphere Reserve Srebarna Lake (North-Eastern Bulgaria). *Lauterbornia*, 49, 99–105.

Pejler, B. (1974) On the rotifer plankton of some East African Lakes. *Hydrobiologia*, 44 (4), 389–396.

Plate, L. (1886) Beiträge zur Naturgeschichte der Rotatorien. *Jenaische Zeitschrift für Naturwissenschaft*, 19, 1–120.

Pliūraitė, V. (2003) Species Diversity of Zooplankton in the Curonian Lagoon in 2001. *Acta Zoologica Lituanica*, 13 (2), 106–113.

Pourriot, R. (1957) Contribution à la connaisance des Rotifères et des Cladocères de la Région parisienne. *Hydrobiologia*, 9, 38–59.

Pourriot, R. (1971) Prospection hydrobiologlque du Lac de Léré et des mares avoisinantes. *Cahiers O.R.S.T.O.M. Série Hydrobiologie*, 5 (2), 171–174.

Pourriot, R. (1975) Rotifères des Antilles. *Cahiers O.R.S.T.O.M. Série Hydrobiologie*, 9, 81–90.

Pradhan, P. & Chakraborty, S.K. (2008) Ecological study of rotifera and its application for biomonitoring of freshwater riverine environment of South West Bengal, India. *Zoological Research in Human Welfare*, 31, 305–321.

Pujin, V., Ratajac, R. & Djukić, N. (1984) Zusamensetzung und dynamic des zooplanktons und der bodenfauna des unteren Theisslaufs. *Tiscia*, 19, 79–87.

Pujin, V., Djukić, N., Maletin, S., Miljanović, B. & Ivanc, A. (1996) Changes of zoocoenotic structure in lower reach of river Tisza. *Tiscia*, 30, 35–38.

Raghunathan, M.B. & Suresh Kumar, R. (2006) Diversity of rotifers of Tamil Nadu. *Records of the Zoological Survey of India*, 106, 67–78.

Raghunathan, M.B. & Valarmathi, K. (2007) Zooplankton investigations from a paddy field in Tamil Nadu. *Records of the Zoological Survey of India*, 107, 55–62.

Redeke, H.C. (1935) *Synopsis van het Nederlandsche zoet- en brakwater-plankton*. Hydrobiologische Club, Amsterdam.

Rico-Martínez, R. & Silva-Briano, M. (1993) Contribution to the knowledge of the Rotifera of Mexico. *Hydrobiologia*, 255–256, 467–474.

Robinson, A.H. & Robinson, P.K. (1971) Seasonal distribution of zooplankton in the northern basin of Lake Chad. *Journal of Zoology*, 163, 25–61.

Rogozin, A.G., Snit’ko, L.V. & Timoshkin, O.A. (2015) Thermoindicator properties of zooplankton species and their measurements. *Water Resources*, 42 (1), 91–97.

Romanescu, G.T., Dinu, C.I., Radu, A.L. & Török, L.I. (2010) Ecologic characterization of the fluviatile limans in the South-West Dobrudja and their economic iImplications (Romania). *Carpathian Journal of Earth and Environmental Sciences*, 5 (2), 25–38.

Rong, S. Segers, H. & Dumont, H.J. Distribution of Brachionidae (Rotifera, Monogononta) in Inner Mongolian Waters. *International Review of Hydrobiology*, 83 (4), 305–310.

Rousselet, C.F. (1906) Contribution to our knowledge of the Rotifera of South Africa. *Journal of the Royal Microscopical Society*, 26, 393–414.

Rylov, W.M. (1927) Über das planktische Vorkommen von Anthophysa steini Senn. *Internationale Revue der gesamten Hydrobiologie und Hydrographie,*18, 75–84.

Saddozai, S., Achakzai, W.M., Masood Z., Kakar, A., Somroo, A.N. & Baloch, W.A. (2015) An Investigation on some New Records of Rotifer species occurs in Manchar Lake of Province Sindh, Pakistan. *Biological Forum – An International Journal,* 7(2), 768–772.

Samraoui, B., Segers, H., Maas, S., Baribwegure, D. & Dumont, H.J. (1998) Rotifera, Cladocera, Copepoda, and Ostracoda from coastal wetlands in northeast Algeria. *Hydrobiologia*, 386, 183–193.

Sancho, V. & Ramia, F. (2008) Data on a relict population of *Emys orbicularis* from Burriana (Castellón, Eastern Spain). [*Revista Española De Herpetología*, 22](http://www.ucm.es/BUCM/compludoc/S/10907/02136686_1.htm), 103–109.

Sanoamuang, L., Segers, H. & Dumont, H.J. (1995) Additions to the rotifer fauna of South-East Asia: new and rare species from North-East Thailand. *Hydrobiologia*, 313/314, 34–45.

Sartory, D.P. (1981) Some planktonic brachionid rotifers from south african impoundments. *Journal of the* *Limnological Society of Southern Africa*, 7 (1), 29–36.

Schmarda, L.K. (1854) Zur Naturgeschichte Ägyptens. *Denkschriften der kaiserlichen Akademie der Wissenschaften, mathematisch-naturwissenschaftliche Classe (Wien)*, 7 (2), 1–28.

Schöll, K. (2006) Changes in rotifer communities regarding to the water-level fluctuations in the floodplain Gemenc, Danube (Hungary). *Opuscula Zoologica Budapest*, 35, 77–81.

Schöll, K. (2009) Diversity of planktonic rotifer assemblages in the water bodies of the Gemenc floodplain (Duna-Dráva National Park, Hungary). *Biologia,* 64/65, 951–958.

Schöll, K. & Kiss, A. (2009) Checklist of the planktonic rotifer fauna in the active floodplain area of the Danube (1843-1806, 1669 and 1437-1489 rkm). *Opuscula Zoologica Budapest*, 40 (2), 63–73.

Schreyer, O. (1920) Die Rotatorien der Umgebung von Beru. *International Review of Hydrobiology*, 9, 311–370.

Semenova, L.M. (2009) The state of zooplankton in the cooler reservoir of the Chernobyl Nuclear Power Plant and in the upper part of the Kiev Reservoir. *Inland Water Biology*, 2 (3), 264–270.

Serpe, F.R., Adloff, C.T., Crispim, M.C. & Rocha, R.M. (2010) Comunidade zooplanctônca em um estuário hipersalino no nordeste do Brasil. *Revista Brasileira de Engenharia de Pesca*, 5 (3), 51–73.

Seymour-Sewell, R.B., 1935. Studies on the bionomics of freshwaters of India. II. On the fauna of the tanks in the Indian Museum Compound and the seasonal changes observed. *Internationale Revue der gesamten Hydrobiologie und Hydrographie,* 31, 203–238.

Sharma, S.P. (1979) Rotifers from West Bengal III. Further studies on the eurotatoria. *Hydrobiologia*, 70, 239–250.

Sharma, S.P. (1980a) Contributions to the rotifer fauna of Orissa, India. *Hydrobiologia*, 70, 225–223.

Sharma, S.P. (1980b) Contributions to the rotifer fauna of Panjab State, India. I. Family Brachionidae. *Hydrobiologia*, 76, 249–253.

Sharma, S.P. & Saksena, D.N. (1984) Form variations in the rotifer Brachionus calyciflorus Palllas from a perennial impoundment in India. *International Review of Hydrobiology*, 69, 747–752.

Sharma, B.K. & Dudani, V.K. (1992) Rotifers from some tropical ponds in Bihar: species composition, similarities and trophic indicators. *Journal of Indian Institute of Sciences*, 72, 121–130.

Sharma, B.K., Sharma, S.S. & Dudani, V.R. (1992) Freshwater rotifers from Darbhanga city, Bihar, India. *Record of Zoological Survey of India*, 91, 431–448.

Sharma, B. K. & Sharma, S. (2000) Freshwater rotifers (Rotifera: Eurotatoria). *Zoological Survey of India- State Fauna series 7: Fauna of Tripura,* 4, 163–224.

Sharma, S. & Sharma, B.K. (2013) *Faunal Diversity of Aquatic Invertebrates of Deepor Beel (A Ramsar site), Assam, Northeast India. Wetland Ecosystem Series, 17*. Zoological. Survey of India, Kolkata.

Sharma, B.K. & Sharma, S. (2014a) Northeast India: An important region with a rich biodiversity of Rotifera. *International Review of Hydrobiology*, 99, 20–37.

Sharma, B.K. & Sharma, S. (2014b) The diversity of Indian Brachionidae (Rotifera: Eurotatoria: Monogononta) and their distribution. *Opuscula Zoologica Budapest*, 45 (2), 165–180.

Shiel, R.J. (1980) Billabongs of the Murray-Darling system. In: Williams, W.D. (ed.), *An Ecological Basis for Water Resource Management*. Australian National University Press, Canberra, 376 – 390.

Shumka, S. (2014) Rotifers in the littoral zone of Lake Shkodra/Skadar (Albania-Montenegro) as a tool for determining water quality. *International Research Journal of Biological Sciences*, 3 (2), 1–6.

Silfverberg, H. (2013) A survey of Rotatoria from Finland. *Memoranda Societatis pro Fauna et Flora Fennica*, 89, 4–16.

Sinha, B. (2014) *Annotated checklist of Indian Rotifera*. ZSI e-publication. Zoological Survey of India, Kolkata, India.

Skorikov, A. (1896) Rotatoria okrestnostei g. Kharkova. *Travaux de la Société des Naturalistes de Charkow*, 30, 207–374.

Sládeček, V. (1955) A note on the occurrence of Hexarthra fennica Levander in Czechoslovakian Oligohaline waters. *Hydrobiologia*, 7, 64–67.

Solanke, M.R. & Dabhade, D.S. (2016) Study of rotifer communities in Upper Morna Reservoir, Medshi, District- Washim, Maharashtra. *International Journal of Applied Research*, 2 (12), 99–102.

Souza-Soares, F., Tundisi, J.G. & Matsumura-Tundisi, T.M. (2011) Checklist of fresh-water Rotifera from São Paulo State, Brazil. *Biota Neotropica*, 11 (1), 516–539.

Spandl, H. (1922) Brachionus pala Ehrbg. var. mucronatus nov. var. *Zoologischer Anzeiger*, 54, 275–275.

Spandl, H. (1923) Zur Kenntnis der Süßwasser-Mikrofauna Vorderasiens. *Annalen des Naturhistorischen Museums in Wien*, 36, 124–149.

Stammer, H.J. (1928) Die Fauna der Ryckmündung, eine Brackwasserstudie. *Zeitschrift für Morphologie und Ökologie der Tiere*, 11, 36–101.

Steinmann, P. & Surbeck, G. (1932) Untersuchungen über das Zooplankton des Rotsees bei Luzern. *Schweizerische zeitschrift fur Hydrologie-Swiss Journal of Hydrology*. 6, 152–215.

Sudzuki, M. (1964) New systematical approach to the Japanese planktonic Rotatoria. *Hydrobiologia*, 23, 1–124.

Sudzuki, M. (1991) The Rotifera from Singapore and Taiwan. *Proceedings of the Japanese Society of Systematic Zoology*, 43, 1–34.

Sudzuki, M. (1992) Seasonal and Local Occurrences of the Rotifera in Southwestern Islands of Japan. *Proceedings of the Japanese Society of Systematic Zoology*, 46, 29–70.

Şundri, M.I. & Gomoiu, M.T. (2009) Qualitative and quantitative structure of zooplankton associations in the Danube thermal discharge area of Nuclear Power Plant Cernavoda. *Geo-Eco-Marina*, 15, 123–130.

Şundri, M.I. (2013) Distribution of Zooplankton Populations Entrained by the Nuclear Power Plant Cernavoda Cooling Water System. In: Neck, R.(ed). *Recent advances in energy, environment, economics and technological innovation. Proceedings of the 4th International Conference on Development, Energy, Environment, Economics (DEEE '13) Proceedings of the 4th International Conference on Communication and Management in Technological Innovation and Academic Globalization (COMATIA '13)* Paris, France October 29–31. WSEAS Press.

Szabó, A. & Kiss Keve, T. (1977) Summer longitudinal section investigations in the Tisza and the Eastern main channel II. Quantitative changes in the zooplankton. *Tiscia*, 12, 85–91.

Tasevska, O., Guseska, D. & Kostoski, G. (2012) Comparison of pelagic rotifer communities in three natural macedonian lakes. *Acta Zoologica Bulgarica*, 4, 159–165.

Tavernini, S., Viaroli, P. & Rossetti, G. (2009). Zooplankton community structure and inter-annual dynamics in two sand-pit lakes with different dredging impact. *International Review of Hydrobiology*, 94 (3), 290–307.

Telesh, I.V. (1996) Species composition of planktonic Rotifera, Cladocera and Copepoda in the littoral zone of Lake Ladoga. *Hydrobiologia*, 322, 181–185.

Tudor, I.M., Ibram, O., Teodorof, L., Burada, A. & Tudor, M. (2016). Present status of zooplankton and benthic invertebrate community structure in Danube delta shallow lakes. *Journal of Environmental Protection and Ecology*, 17 (1), 228–236.

Vadadi-Fülöp, C.S., Mészáros, G., Jablonszky, G.Y., Hufnagel, L. (2007) Ecology of the Ráckeve-Soroksár Danube - A review. *Applied Ecology and Environmental Research,* 5 (1), 133–163.

Vaidya, S.R. & Yadav, U.K.R. (2008) Ecological study on zooplankton of some fresh water bodies of Kathmandu Valley with reference to water quality. *Journal of the Natural History Museum*, 23, 1–11.

Välikangas, I. (1926) Planktologische Untersuchungen im Hafengebiet von Helsingfors. *Acta Zoologica Fennica*, 1, 1–298

Van Oye, P. (1924). Zur Biologie des Potamoplanktons auf Java. *Internationale Revue der gesamten Hydrobiologie und Hydrographie,* 12, 48–59.

Vásquez, E. & Rey, J. (1989). A longitudinal study of zooplankton along the Lower Orinoco River and its Delta (Venezuela). *Annales de Limnologie*, 25, 107–120.

Velasco, J.L., Αlvarez, M., Colomer, M. & Rubio, A. (1996) El «Mar de Ontígola» (Madrid): Características limnologicas. *Anales de Biología*, 21 (10), 93–104.

Velasco, J.L. (2006) Rotíferos de la comunidad de Madrid. *Graellsia*, 62, 33–42.

Vezhnavets, V.V., Malatkou, D.V. & Arbačiauskas, K. (2014) Zooplankton community. *Zoology and Ecology*, 24 (2), 108–127.

Virro, T. (1995) The genus Polyarthra in Lake Peipsi. *Hydrobiologia,* 313/314, 351–357.

Virro, T. & Haberman, J. (2005) Annotated list of rotifers of Lake Võrtsjärv. [*Proceedings of the Estonian Academy of Sciences, Biology and Ecology*](https://books.google.gr/books?id=1IcwnNcGB0oC&pg=PA290&lpg=PA290&dq=Proc.+Estonian+Acad.+Sci.+Biol.+Ecol.,&source=bl&ots=A__vdwKybb&sig=19iAHyUde-XxZNgRBYyJiVvgI_Y&hl=en&sa=X&ved=0ahUKEwj4kvz20fzTAhVEiCwKHZatBoUQ6AEIOjAD), 54 (1), 53–66.

Watzka, M. (1928) Die Rotatorienfauna der Cakowitzer Zuckerfabriksteiche und Versuche über das Auftreten von Rotatorien-Männchen und über die Entwicklungszeit der Dauereier. *Internationale Revue der gesamten Hydrobiologie und Hydrographie*, 19, 430–451.

[Wawrik](http://www.zobodat.at/personen.php?id=18896), F. (1960) Waldviertler Fischteiche II - Die Jaidhof-Teiche. - *Sitzungsberichte Der Akademie Der Wissenschaften Mathematisch-Naturwissenschaftliche Klasse,* 169, 341–381.

Wawrik, F. (1972) Vergleichende braunwasser-teichstudien im niederosterreichischen Waldviertel. *Hydrobiologia,* 39 (1), 17–82.

Weber, E.F. (1898) Faune Rotatorienne du bassin du Léman. *Revue Suisse de Zoologie*, 5, 263–785.

Weisse, J.F. (1845) Beschreibung einiger neuer Infusorien, welche in stehenden Wässern bei St. Petersburg vorkommen. Beschreibung einiger neuer Infusorien, welche in stehenden Wässern bei St. *Petersburg vorkommen. Bulletin de la Classe Physico-Mathématique de l'Académie Impériale des Sciences de St.-Pétersbourg*, 4, 138–143.

Wierzejski, A. (1891) Liste de rotifères observes en Galicie (Autriche-Hongrie). *Bulletin de la Société zoologique de France*, 16, 49–52.

Wijetunge, D.S. & Ranatunga, R.R.M.K.P. (2012) Zooplankton Assemblage in Hambanota Port and Adjacent Coastal Waters of Sri Lanka. *Vidyodaya Journal of Science*, 17, 9–23.

Woltereck, R., Tressler, W.S. & Bunag, D.M. (1941) Die Seen und Inseln der "Wallacea"-Zwishenregion und ihre endemische Tierwelt. Zweiter Teil: Inseln und Seen der Philippinen. *Internationale Revue der gesamten Hydrobiologie und Hydrographie*, 30, 37–76.

Yamamoto, K. (1960) Plankton rototorian Japanese Inland. *Hydrobiologia*, 16 (4), 364–41.

Yermolaeva, N.I. (2013) Some Results of Studying Zooplankton in Lakes of Northern Kazakhstan. *Arid Ecosystems*, 3 (4), 263–275.

Yermolaeva, N.I. (2015) Zooplankton and water quality of the Ishim River in Northern Kazakhstan. *Arid Ecosystems*, 5 (3), 176–187.

Yermolaeva, N.I., Zarubina, E.Y., Romanov, R.E., Leonova, G.A & Puzanov, A.V. (2016) Hydrobiological conditions of sapropel formation in Lakes in the South of Western Siberia. *Water Resources*, 43 (1), 129–140.

Zacharias, O. (1898) *Forschungsberichte aus der Biologischen Station zu Plön*. Nägele, Stuttgart

Zarfdjian, M., Vranovský, M. & Economidis, P.S. (1990) Les invertébrés planctoniques du lac Volvi (Macédoine, Grèce). *International Revue der Gesamten Hydrobiologie*, 75, 403–412.

Zernov, S.A. (1901) zametki o zhivotnom planktone rek shoshmy i vjatki malmyzhskogo ujezda vjatskoj gub. izvestija imperatorskogo obshchestva lyubitelei jestestvoznanija. *Antropologii i Etnografii pri Moskovskom Universitjete*, 98 (2), 25–36.

Zhang, S.Y., Zhou, Q.H., Xu,. D., Lin, J.D., Cheng, S.P. & Wu, Z.B. (2010) Effects of sediment dredging on water quality and zooplankton community structure in a shallow of eutrophic lake. *Journal of Environmental Sciences-China*, 22 (2), 218–224.

Zinevici, V., Parpală, L., Florescu, L. & Moldoveanu, M. (2013) The zooplankton of the danube-black sea canal in the first two decades of the ecosystem existence. *Travaux du Muséum National d’Histoire Naturelle «Grigore Antipa»*, 56 (2), 227–251.
