# Supplementary material for: Reverse taxonomy applied to the Brachionus calyciflorus cryptic species complex: Morphometric analysis confirms species delimitations revealed by molecular phylogenetic analysis and allows the (re)description of four species
Source: PLoS One. 2018 Sep 20;13(9):e0203168. doi: 10.1371/journal.pone.0203168 (PMC6147415; doi:10.1371/journal.pone.0203168)
Supplement: S1 Table — (DOCX) [file pone.0203168.s002.docx]

**S1 Table.** **Amount of morphometric variation explained by species identity for each pair of the investigated species.** Percentages refer to adjusted R^2^-values of redundancy analyses. *: p< 0.05; **: p< 0.01; ***: p< 0.001.

|  | A | B | C |
| --- | --- | --- | --- |
| B | 43%*** |  |  |
| C | 55%*** | 24%** |  |
| D | 34%*** | 19%** | 38%*** |
