# Supplementary material for: Reverse taxonomy applied to the Brachionus calyciflorus cryptic species complex: Morphometric analysis confirms species delimitations revealed by molecular phylogenetic analysis and allows the (re)description of four species
Source: PLoS One. 2018 Sep 20;13(9):e0203168. doi: 10.1371/journal.pone.0203168 (PMC6147415; doi:10.1371/journal.pone.0203168)
Supplement: S2 Table — (DOCX) [file pone.0203168.s003.docx]

**S2 Table.** **Summary statistics (minimum, maximum, mean and standard error, in μm) for the selection of morphometric traits measured on individuals of the four species.** * zero values recorded only for two individuals

|  | A |  |  |  | B |  |  |  | C |  |  |  | D |  |  |  |
| --- | --- | --- | --- | --- | --- | --- | --- | --- | --- | --- | --- | --- | --- | --- | --- | --- |
|  | min | max | mean | St. Error | min | max | mean | St. Error | min | max | mean | St. Error | min | max | mean | St. Error |
| s | 112.10 | 214.67 | 149.96 | 1.27 | 94.51 | 176.35 | 133.31 | 0.91 | 77.58 | 182.59 | 124.19 | 1.28 | 115.18 | 182.34 | 153.70 | 1.12 |
| c | 121.39 | 247.58 | 186.07 | 1.46 | 106.88 | 217.44 | 158.48 | 1.18 | 89.65 | 203.91 | 140.11 | 1.63 | 131.28 | 200.05 | 166.07 | 1.28 |
| e | 43.84 | 92.62 | 67.16 | 0.78 | 29.54 | 74.20 | 49.82 | 0.47 | 25.17 | 69.82 | 43.05 | 0.69 | 34.62 | 72.31 | 52.57 | 0.57 |
| b | 87.09 | 196.00 | 146.58 | 1.43 | 76.31 | 183.92 | 115.72 | 1.10 | 82.29 | 175.45 | 122.18 | 1.38 | 49.02 | 158.17 | 126.83 | 1.28 |
| o | 32.71 | 67.16 | 48.54 | 0.44 | 28.91 | 64.56 | 40.66 | 0.40 | 28.02 | 64.87 | 40.94 | 0.41 | 25.79 | 54.73 | 43.43 | 0.43 |
| h | 33.44 | 59.86 | 46.71 | 0.42 | 22.23 | 56.48 | 38.01 | 0.40 | 15.00 | 60.34 | 37.81 | 0.59 | 30.58 | 60.45 | 44.05 | 0.53 |
| k | 52.41 | 86.21 | 66.61 | 0.57 | 32.77 | 73.83 | 51.89 | 0.43 | 31.14 | 68.89 | 49.20 | 0.52 | 40.77 | 70.96 | 57.24 | 0.50 |
| j | 46.32 | 98.38 | 71.25 | 0.77 | 33.17 | 75.56 | 53.38 | 0.48 | 25.15 | 74.05 | 46.55 | 0.74 | 38.07 | 73.77 | 55.98 | 0.60 |
| t | 4.43 | 15.90 | 8.82 | 0.16 | 4.37 | 19.29 | 10.78 | 0.15 | 3.26 | 14.88 | 7.52 | 0.15 | 4.48 | 18.45 | 8.86 | 0.19 |
| i | 106.45 | 178.59 | 146.73 | 1.09 | 73.71 | 179.25 | 118.37 | 0.99 | 75.24 | 163.55 | 109.38 | 1.34 | 92.79 | 147.91 | 130.33 | 0.91 |
| p | 29.71 | 56.70 | 44.78 | 0.41 | 16.87 | 48.14 | 30.78 | 0.36 | 11.73 | 63.24 | 33.23 | 0.90 | 17.89 | 58.44 | 33.85 | 0.91 |
| v | 8.00 | 24.31 | 14.92 | 0.24 | *0.00 | 28.53 | 14.35 | 0.25 | 0.00 | 32.41 | 10.62 | 0.88 | 0.00 | 35.42 | 15.68 | 1.00 |
| x | 11.37 | 24.45 | 16.47 | 0.21 | 9.68 | 29.00 | 18.02 | 0.19 | 6.04 | 21.19 | 12.95 | 0.24 | 11.93 | 27.87 | 19.70 | 0.26 |
| z | 51.44 | 89.44 | 72.91 | 0.53 | 36.88 | 88.83 | 58.68 | 0.50 | 35.26 | 75.82 | 53.81 | 0.65 | 46.51 | 76.27 | 65.24 | 0.48 |
| q | 20.08 | 39.71 | 29.03 | 0.29 | *0.00 | 42.96 | 28.97 | 0.34 | 0.00 | 46.14 | 16.00 | 1.32 | 0.00 | 52.80 | 27.29 | 1.61 |
| r | 14.75 | 40.66 | 27.40 | 0.38 | 15.95 | 49.17 | 29.33 | 0.34 | 13.47 | 38.25 | 22.50 | 0.40 | 21.33 | 45.50 | 35.17 | 0.43 |
| sta | 146.14 | 285.38 | 214.07 | 1.72 | 115.64 | 283.51 | 185.85 | 1.45 | 121.26 | 271.29 | 176.75 | 2.04 | 167.35 | 246.27 | 212.58 | 1.37 |
