# Supplementary material for: Reverse taxonomy applied to the Brachionus calyciflorus cryptic species complex: Morphometric analysis confirms species delimitations revealed by molecular phylogenetic analysis and allows the (re)description of four species
Source: PLoS One. 2018 Sep 20;13(9):e0203168. doi: 10.1371/journal.pone.0203168 (PMC6147415; doi:10.1371/journal.pone.0203168)
Supplement: S3 Table — Classification functions of the stepwise discriminant analysis performed on species ‘A’, ‘B’, ‘C’, and ‘D’. (DOCX) [file pone.0203168.s004.docx]

**S3 Table. Classification functions of the stepwise discriminant analysis performed on species A, B, C, and D.**

| Measurement | A | B | C | D |
| --- | --- | --- | --- | --- |
| s | -1.647 | -.778 | .231 | 2.970 |
| c | 3.551 | 1.339 | -1.438 | -4.508 |
| e | 1.994 | .137 | -1.399 | -.587 |
| b | .633 | -.593 | 1.563 | -1.699 |
| o | -1.369 | .142 | 1.061 | -.173 |
| h | -3.432 | -.830 | 1.990 | 2.682 |
| k | 3.410 | -.959 | -.945 | -.725 |
| j | 2.037 | .773 | -1.383 | -1.844 |
| t | -1.126 | 1.187 | -.087 | -.849 |
| i | 3.927 | -.454 | .101 | -3.650 |
| p | 2.134 | 1.212 | -3.226 | -.291 |
| v | -4.242 | -1.520 | 2.075 | 4.756 |
| x | -1.528 | .966 | -.765 | .921 |
| z | -.007 | -2.036 | 1.266 | 2.106 |
| q | 6.279 | 3.177 | -5.349 | -5.707 |
| r | -3.764 | .649 | -.592 | 3.765 |
| sta | -3.123 | -.739 | 1.038 | 3.452 |
| Constant | -8.949 | -2.627 | -4.089 | -6.285 |
