# Supplementary material for: Reverse taxonomy applied to the Brachionus calyciflorus cryptic species complex: Morphometric analysis confirms species delimitations revealed by molecular phylogenetic analysis and allows the (re)description of four species
Source: PLoS One. 2018 Sep 20;13(9):e0203168. doi: 10.1371/journal.pone.0203168 (PMC6147415; doi:10.1371/journal.pone.0203168)
Supplement: S4 Table — Classification functions of the stepwise discriminant analysis performed on species ‘B’, ‘C’, and ‘D’. (DOCX) [file pone.0203168.s005.docx]

**S4 Table. Classification functions of the stepwise discriminant analysis performed on species B, C, and D.**

| Measurement | B | C | D |
| --- | --- | --- | --- |
| s | -.987 | -.167 | 3.176 |
| c | 1.052 | -1.614 | -5.076 |
| b | -.565 | 1.260 | -2.187 |
| o | -.215 | .650 | -.405 |
| h | -1.040 | 1.581 | 2.466 |
| j | .316 | -3.456 | -2.864 |
| t | 1.195 | -.161 | -.871 |
| v | -1.984 | 3.674 | 4.032 |
| x | .489 | -.322 | .778 |
| q | 2.529 | -3.948 | -4.537 |
| r | .479 | -.438 | 3.184 |
| sta | -.981 | .868 | 2.915 |
| Constant | -2.148 | -3.470 | -5.723 |
